# Increased Prediction Ability in Norway Spruce Trials Using a Marker X Environment Interaction and Non-Additive Genomic Selection Model

**DOI:** 10.1093/jhered/esz061

**Published:** 2019-10-20

**Authors:** Zhi-Qiang Chen, John Baison, Jin Pan, Johan Westin, Maria Rosario García Gil, Harry X Wu

**Affiliations:** 1 Umeå Plant Science Centre, Department of Forest Genetics and Plant Physiology, Swedish University of Agricultural Sciences, Umeå, Sweden; 2 Skogforsk, Sävar, Sweden; 3 Beijing Advanced Innovation Centre for Tree Breeding by Molecular Design, Beijing Forestry University, Beijing, China; 4 CSIRO National Collection Research Australia, Black Mountain Laboratory, Canberra, ACT, Australia

**Keywords:** dominance, epistasis, exome capture, *Picea abies* (L.) Karst

## Abstract

A genomic selection study of growth and wood quality traits is reported based on control-pollinated Norway spruce families established in 2 Northern Swedish trials at 2 locations using exome capture as a genotyping platform. Nonadditive effects including dominance and first-order epistatic interactions (including additive-by-additive, dominance-by-dominance, and additive-by-dominance) and marker-by-environment interaction (M×E) effects were dissected in genomic and phenotypic selection models. Genomic selection models partitioned additive and nonadditive genetic variances more precisely than pedigree-based models. In addition, predictive ability in GS was substantially increased by including dominance and slightly increased by including M×E effects when these effects are significant. For velocity, response to genomic selection per year increased up to 78.9/80.8%, 86.9/82.9%, and 91.3/88.2% compared with response to phenotypic selection per year when genomic selection was based on 1) main marker effects (M), 2) M + M×E effects (A), and 3) A + dominance effects (AD) for sites 1 and 2, respectively. This indicates that including M×E and dominance effects not only improves genetic parameter estimates but also when they are significant may improve the genetic gain. For tree height, Pilodyn, and modulus of elasticity (MOE), response to genomic selection per year improved up to 68.9%, 91.3%, and 92.6% compared with response to phenotypic selection per year, respectively.Subject Area: Quantitative genetics and Mendelian inheritance

Genomic selection (GS) is a breeding method that uses a dense set of genetic markers to accurately predict the genetic merit of individuals (Meuwissen et al. 2001) and it has been incorporated into animal breeding for many years (Van Eenennaam et al. 2014). Simulated studies have also shown that including dominance could increase the predictive ability (PA) (Nishio and Satoh 2014) and result in a higher genetic gain in crossbred population when the dominance variance and heterosis are large and over-dominance is present (Zeng et al. 2013). In livestock, accounting for dominance in GS has improved genomic evaluations of dairy cows for fertility and milk production traits (Aliloo et al. 2016). In tree species, GS studies have been implemented in several breeding programs, but these studies mostly focused on additive effects in several commercially important conifer species, such as loblolly pine (*Pinus taeda* L.), maritime pine (*Pinus pinaster* Ait.), Norway spruce (*Picea abies* (L.) Karst.), white spruce (*Picea glauca* (Moench) Voss) and hardwood eucalypt species (Resende et al. 2012a, 2012b; Tan et al. 2017; Chen et al. 2018). The nonadditive contributions have also been estimated in several studies (Muñoz et al. 2014; Bouvet et al. 2016; de Almeida Filho et al. 2016; Gamal El-Dien et al. 2016; Tan et al. 2018).

Several recent studies show dominance and epistasis may be confounded with the additive effects in both pedigree-based relationship matrix models (Gamal El-Dien et al. 2018) and genomic-based relationship matrix models (Tan et al. 2018). In the conventional pedigree-based genetic analysis, estimates of different genetic components such as additive, dominance, and epistatic variances need full-sib family structure or full-sib family structure plus clonally replicated tests (Mullin and Park 1992). For most tree species, only a few reliable estimates for the nonadditive variation have been reported based on pedigree-based relationship (Isik et al. 2003, 2005; Baltunis et al. 2007; Weng et al. 2008; Wu et al. 2008), especially for wood quality traits (Wu 2018).

Significant genotype-by-environment (G×E) interaction is commonly observed among the different deployment zones for growth traits in Norway spruce (Kroon et al. 2011; Chen et al. 2014, 2017). Literature also supports the importance of predicting nonadditive effects including dominance and epistasis in tree breeding (Wu et al. 2016) and in clonal forestry programs (Wu 2018). In a previous study (Chen et al. 2018), we used 2 full-sib family trials to study GS efficiency based on additive effects and different sampling strategies. Here, we extend our study to examine nonadditive genetic effects using the genomic matrix and to explore marker-by-environment interaction (M×E) effects on GS. The aims of the study were to 1) estimate and compare the nonadditive genetic variances estimated from the average numerator relationship A-matrix (i.e. the expected theoretical relationships) and the realized genomic relationship G-matrix (i.e. the observed relationships); 2) evaluate the PA of different M×E models; 3) assess the PA of the models including nonadditive effects; 4) evaluate change in the ranking of breeding values when models include the nonadditive and M×E effects; and 5) assess genetic gain per year when M×E and dominance effects are included in the GS and phenotypic selection (PS) models.

## Materials and Methods

### Sampling of Plant Material and Genotyping

In all, 1,370 individuals were selected from two 28-year-old control-pollinated (full-sib) progeny trials. The progeny trials consist of the same 128 families generated through a partial diallel mating design involving 55 parents originating from Northern Sweden. Progenies were raised in the nursery for 1 year at Sävar, and the trials were established in 1988 by Skogforsk in Vindeln (64.30°N, 19.67°E, altitude: 325 m) and in Hädanberg (63.58°N, 18.19°E, altitude: 240 m).

A completely randomized design without designed pre-blocking was used in the Vindeln trial (site 1), which was divided into 44 post-blocks based on the terrain. Each rectangular block has 60 trees (6 ×10) with expected 60 families at a spacing of 1.5 m × 2.0 m. The same design was also used in the Hädanberg trial (site 2) with 44 post-blocks. But for the purpose of demonstration, there was an extra block with 47 plots, each plot with 16 trees (4×4) planted in site 2. Based on the spatial analysis, in the final model, the 47 plots were combined into 2 big post-blocks.

### Phenotyping

The tree height was measured in 2003 at the age of 17 years. Solid-wood quality traits including Pilodyn penetration (Pilodyn) and acoustic velocity (velocity) were measured in October 2016. Pilodyn penetration, a surrogate for the trait of wood density, was measured using a Pilodyn 6J Forest (PROCEQ, Zurich, Switzerland) with a 2.0 mm diameter pin, without removing the bark. Velocity, closely related to microfibril angle (MFA) in Norway spruce (Chen et al. 2015), was determined using a Hitman ST300 (Fiber-gen, Christchurch, New Zealand). By combining the Pilodyn and velocity data, indirect modulus of elasticity (MOE) was estimated using the equation developed in the study by Chen et al. (2015).

### Genotyping

Buds and the first-year fresh needles from 1370 control-pollinated progeny trees and their 46 unrelated parents were sampled and genotyped using the Qiagen Plant DNA extraction protocol (Qiagen, Hilden, Germany) and DNA quantification was undertaken using the Qubit® ds DNA Broad Range Assay Kit (Oregon, USA). The 46 parents were sampled in a grafted archive at Skogforsk, Sävar (63.89°N, 20.54°E) and in a grafted seed orchard at Hjssjö (63.93°N, 20.15°E). Probe design and evaluation are described by Vidalis et al. (2018). Sequence capture was performed using the 40 018 probes previously designed and evaluated for the material (Vidalis et al. 2018) and samples were sequenced to an average depth of 15x on an Illumina HiSeq 2500 platform. The details of SNPs calling, filtering, quality control, and imputation for these data can be found in Chen et al. (2018). Finally, 116,765 SNPs were kept for downstream analysis.

### Variance Component and Heritability Models

The variance components and breeding values (BVs) for the genotypes of each trait in the 2 trials were estimated by using the best linear unbiased prediction (BLUP) method in 3 univariate models that included either additive (A), both additive and dominance (AD) or additive, dominance, and epistasis genetic effects (ADE) as mentioned below. In practice, pedigree-based models (ABLUP) had only 2 models because it is not possible to estimate the epistatic effect in full-sib progeny trials without replicates for each genotype.

### Pedigree-Based and Genomic-Based Models

Five models were used to partition the genetic variance into additive, dominance, and epistatic variances.

For the pedigree-based model with additive effect only (ABLUP-A):

$$\begin{array}{l} y=X\beta +Wb+Za+\varepsilon \end{array}$$ (1)

For the full pedigree-based model with both additive and dominance effects (ABLUP-AD):

$$\begin{array}{l} y=X\beta +Wb+Za+Z_{1}d+\varepsilon \end{array}$$ (2)

For the genomic-based model with additive effect only (GBLUP-A):

$$\begin{array}{l} y=X\beta +f\;i+Wb+Z_{2}a_{1}+\varepsilon \end{array}$$ (3)

For the genomic-based model with both additive and dominance effects (GBLUP-AD):

$$\begin{array}{l} y=X\beta +f\;i+Wb+Z_{2}a_{1}+Z_{3}d_{1}+\varepsilon \end{array}$$ (4)

For the full genomic-based model with additive, dominance, and epistatic effects (GBLUP-ADE):

$$\begin{array}{l} y=X\beta +f\;i+Wb+Z_{2}a_{1}+Z_{3}d_{1}+Z_{4}e_{aa}+Z_{5}e_{ad}+Z_{6}e_{dd}+\varepsilon \end{array}$$ (5)

where ***y*** is the vector of phenotypic observations of a single trait; β is the vector of fixed effects, including a grand mean and site effects, *i is* the inbreeding depression parameter per unit of inbreeding, *b* is the vector of random post-block within site effects, a and $a_{1}$ are the vectors of random additive effects in ABLUP and GBLUP models, respectively, *d* and $d_{1}$are the vectors of random dominance effects in equations [2], [4], and [5], respectively, $\;e_{aa},\;e_{ad},\;and\;e_{dd}$ are the vectors of the random additive-by-additive epistatic effects, additive-by-dominance epistatic effects, and dominance-by-dominance epistatic effects in equation (5), ε is the random residual effect. ***X***, ***W***, ***Z*, *Z***_1_, ***Z***_2_, ***Z***_3_, ***Z***_4_**, *Z***_1_, and ***Z***_6_ are the incidence matrices for β, b, a, d, $a_{1}$, $d_{1}$, $e_{aa}$, $e_{ad}$, and $e_{dd}$, respectively. *f* is a vector of genomic inbreeding coefficients based on the proportion of homozygous SNPs. Although Xiang et al. (2016) and Vitezica et al. (2013) proved that including genomic inbreeding as a covariate is necessary to obtain correct estimates of dominance and epistatic variances, the inbreeding depression term (*fi*) in equation (3–5) were excluded in the final model because it is not significant for all the traits. The random post-block effects (b) were assumed to follow

$$\begin{array}{l} b∼N\left(0,\;\left[\begin{array}{ll} \sigma_{b1}^{2} & 0\\ 0 & \sigma_{b2}^{2} \end{array}\right]⊗I\right), \end{array}$$

where *I* is the identity matrix, $\sigma_{b1}^{2}$ and $\sigma_{b1}^{2}$ are the variance components of random post-block in site 1 and site 2, respectively, and ⊗ is the Kronecker product operator. The random additive effects (a) in equations (1) and (2) were assumed to follow $a∼N\left(0,VCOV_{a}⊗A\right)$, where *A* is the pedigree-based additive genetic relationship matrix and $VCOV_{a}$ is the general case of additive variance and covariance structure in Table 1. The random dominance effects (*d*) in equation [2] were assumed to follow $d∼N\left(0,VCOV_{d}⊗D\right)$, where *D* is the pedigree-based dominance relationship matrix and $VCOV_{d}$ is the general case of dominance variance and covariance structure. The $a_{1}$ in equations (3–5) is the vector of random additive effects in genomic-based models, following $a_{1}∼N(0,VCOV_{a}⊗G_{a})$, where $G_{a}$ is the genomic-based additive genetic relationship matrix, $VCOV_{a}$ is the general case of additive variance and covariance structure in Table 1. The $d_{1}$ in equations (4) and (5) is the vector of random dominance effects following $d_{1}∼N(0,\;VCOV_{d}⊗G_{d})$, where $G_{d}$ is the genomic-based dominance genetic relationship matrix, $VCOV_{d}$ is the general case of dominance variance and covariance structure in Table 1. The $e_{aa},\;e_{ad},\;and\;e_{dd}$ are the vectors of the random additive-by-additive epistatic effects, additive-by-dominance epistatic effects, and dominance-by-dominance epistatic effects following $e_{aa}∼\;N(0,G_{aa}\sigma_{aa}^{2})$, $e_{ad}∼\;N(0,G_{ad}\sigma_{ad}^{2})$, and $e_{dd}∼\;N(0,G_{dd}\sigma_{dd}^{2})$, respectively. $G_{aa}$, $G_{ad}$, and $G_{dd}$ are the genomic-based additive-by-additive, additive-by-dominance, and dominance-by-dominance epistatic relationship matrices, respectively. The residual *e* was assumed to follow

**Table 1.** Six variance and covariance structures examined for the additive, dominance, and epistatic effects in 2 pedigree-based models and 3 genomic-based models.

Structure	No. of parameters	Description
IDEN	1	Identity
DIAG	n	Diagonal
CS	2	Compound symmetry
CS+DIAG	1 + *n*	Compound symmetry with heterogeneous variance
US	*n*(*n* + 1)/2	Unstructured
FAMK	1 + (*k* + 1)*n*	Factor analytic with the main marker/genetic term and k factors

*n* is the number of sites. *k* is the number of factors.

$$\begin{array}{l} \varepsilon ∼N\left(0,\left[\begin{array}{ll} I_{n1}\Sigma_{e1}^{2} & \;0\\ \;0 & \;I_{n2}\Sigma_{e2}^{2} \end{array}\right]\right), \end{array}$$

where $\Sigma_{e1}^{2}$ and $\Sigma_{e2}^{2}$ are the residual variances for site 1 and site 2, respectively, $I_{n1}$ and $I_{n2}$ are identity matrices, and n1 and n2 are the number of individuals at each site. In theory, all variance–covariance structures in Table 1 could be used for additive, dominance, and epistatic effects in equations (1)–(5).

The pedigree-based additive (*A*) and dominance (*D*) relationship matrices were constructed based on information from pedigrees. The diagonal elements (*i*) of the *A* were calculated as $A_{ii}\;=1+f_{i}=1+A_{gh}/2$, where g and h are the ith individual’s parents, while the off-diagonal element is the relationship between individuals ith and jth calculated as $A_{ij}=A_{ji}=(A_{jg}+A_{jh})/2$ (Mrode and Thompson 2005). In the *D* matrix, the diagnonal elements were all one ($D_{ii}=1$), while the off-diagonal elements between the individual *i*th and *j*th can be calculated as $D_{ij}=(A_{gk}A_{hl}+A_{gl}A_{hk})/4$, where *g* and *h* are the parents of the *i*th individual and *k* and *l* are the parents of the *j*th individual. *A* relationship matrix was produced using ASReml 4.1 (Gilmour et al. 2015) or ASReml-R package (Butler et al. 2009). A *D* relationship matrix was produced using kin function in the synbreed package in R (Wimmer et al. 2012).

The genomic-based additive ($G_{a}$) and dominance ($G_{d}$) relationship matrices were constructed based on genome-wide exome capture data as described by VanRaden (2008) for $G_{a}$ and by Vitezica et al. (2013) for $G_{d}$:

$$\begin{array}{l} G_{a}=\frac{ZZ^{\prime }}{\overset{m}{\underset{j=1}{\sum }}2p_{i}q_{i}} \end{array}$$

$$\begin{array}{l} G_{d}=\frac{WW^{\prime }}{\overset{m}{\underset{i=1}{\sum }}{\left(2p_{i}q_{i}\right)}^{2}} \end{array}$$

where m is the total number of SNPs; the elements of ***Z*** are equal to $-2p_{i}$, $q_{i}-p_{i}$, and $2q_{i}$ for **aa**, **Aa**, and **AA** genotypes, respectively, with $p_{i}$ and $q_{i}$being the allele frequency of **A** and **a** alleles at marker i in the population. For the dominance matrix $G_{d}$, **aa, Aa**, and **AA** genotypes in W were coded as $-2p_{i}^{2}$, $2p_{i}q_{i}$, and $-2q_{i}^{2}$, respectively. Based on the paper of Vitezica et al. (2013), the method guarantees the absence of confounding between $G_{a}$ and $G_{d}$ and could be directly compared to the pedigree-based *A* and *D*.

The relationship matrices due to the first-order epistatic interactions were computed using the Hadamard product (cell by cell multiplication, denoted #) and trace (*tr*) (Vitezica et al. 2013). In the pedigree-based model, the additive-by-additive terms are calculated as $P_{aa}=\left[\left(A\#A\right)/\left(tr(A\#A)/n\right)\right]$, additive-by-dominance terms as $P_{ad}=\;\;\;\left[\left(A\#D\right)/\left(tr(A\#D)/n\right)\right]$, and dominance-by-dominance terms as $P_{dd}=\left[\left(D\#D\right)/\left(tr(D\#D)/n\right)\right]$. In genomic-based relationship matrix models: additive-by-additive terms $are\;G_{aa}=\left[\left(G_{a}\#G_{a}\right)/\left(tr(G_{a}\#G_{a})/n\right)\right]$, additive-by-dominance terms are $G_{ad}=\left[\left(G_{a}\#G_{d}\right)/\left(tr(G_{a}\#G_{d})/n\right)\right]$, and dominance-by-dominance terms are $G_{dd}=\left[\left(G_{d}\#G_{d}\right)/\left(tr(G_{a}\#G_{d})/n\right)\right]$.

### Different Variance–Covariance Structures

To partition, predict, and validate G×E interactions in additive (*a*), dominance (*d*), epistatic effects ($e_{aa}$, $e_{ad}$, and $e_{dd})$, 6 types of the different variance and covariance structures (Table 1) including: 1) identity (IDEN), 2) diagonal (DIAG), 3) compound symmetry (CS), 4) compound symmetry with heterogeneous variance (CS+DIAG), 5) unstructured (US), and 6) factor analytic with the main marker/genetic term and k factors (FAMK), could be fitted for any of the additive, dominance, and epistasis effects in equation (1)–(5). The CS+DIAG, US, and FAMK structures are the same in any two-sites multi-environment trial (MET) model (Oakey et al. 2016), except that the models may have a slightly convergent difference. When MET models with more than 2 sites were used, the models with FAMK structure may be better than those with CS+DIAG and US (Oakey et al. 2016). We therefore presented only the FAMK model in the latter. The additive variance–covariance structures of IDEN, DIAG, CS, and FAMK are, , respectively,

$$\begin{array}{l} \left[\begin{array}{cc} \sigma_{a}^{2} & 0\\ 0 & \sigma_{a}^{2} \end{array}\right],\;\left[\begin{array}{cc} \sigma_{a1}^{2} & 0\\ 0 & \sigma_{a2}^{2} \end{array}\right],\;\left[\begin{array}{cc} \sigma_{a}^{2} & \sigma_{a12}\\ \sigma_{a21} & \sigma_{a}^{2} \end{array}\right],\;and\;\left[\begin{array}{cc} \sigma_{a1}^{2} & \sigma_{a12}\\ \sigma_{a12} & \sigma_{a2}^{2} \end{array}\right] \end{array}$$

The dominance variance structures of IDEN, DIAG, CS, and FAMK are , respectively,

$$\begin{array}{l} \left[\begin{array}{cc} \sigma_{d}^{2} & 0\\ 0 & \sigma_{d}^{2} \end{array}\right],\;\left[\begin{array}{cc} \sigma_{d1}^{2} & 0\\ 0 & \sigma_{d2}^{2} \end{array}\right],\;\left[\begin{array}{cc} \sigma_{d}^{2} & \sigma_{d12}\\ \sigma_{d21} & \sigma_{d}^{2} \end{array}\right],\;and\;\left[\begin{array}{cc} \sigma_{d1}^{2} & \sigma_{d12}\\ \sigma_{d12} & \sigma_{d2}^{2} \end{array}\right] \end{array}$$

In this study, the result of epistasis effects is shown only with the variance and covariance structure IDEN because of the small amount of the total genetic variance. $\sigma_{a}^{2}$ and $\sigma_{d}^{2}$ are the additive and dominance variances if homogenous variance structures were used. $\sigma_{a1}^{2}$, $\sigma_{a2}^{2}$, and $\sigma_{a12}$ are the additive variances for site 1, site 2 and the additive covariance between sites 1 and 2, respectively. $\sigma_{d1}^{2}$, $\sigma_{d2}^{2}$, and $\sigma_{d12}$ are dominance variances for site 1, site 2 and dominance covariance between sites 1 and 2.

### Heritability

Under the above models, the narrow-sense heritability can be estimated as $h^{2}=\sigma_{a}^{2}/\sigma_{p}^{2}$, the dominance to total variance ratio as $d^{2}=\sigma_{d}^{2}/\sigma_{p}^{2}$, the epistatic to the total variance ratio as $i^{2}=\sigma_{i}^{2}/\sigma_{p}^{2}$ and the broad-sense heritability as $H^{2}=\sigma_{g}^{2}/\sigma_{p}^{2}$, where $\sigma_{g}^{2}=\sigma_{a}^{2}+\sigma_{d}^{2}+\sigma_{aa}^{2}+\sigma_{ad}^{2}+\sigma_{dd}^{2}$ and $\sigma_{i}^{2}=$$\sigma_{aa}^{2}+\sigma_{ad}^{2}+\sigma_{dd}^{2}$. Broad-sense heritability for the ABLUP-AD model was estimated as $H^{2}=(\sigma_{a}^{2}+\sigma_{d}^{2})/\sigma_{p}^{2}$ as epistatic effects could not be estimated.

#### To partition and Predict Gxe Interaction and Dominance in Cross-Validation

To compare the predictive ability of models with and without a G×E interaction term in additive effects, a single-site model without specifying the G×E interaction (i.e. ABLUP-AD and GBLUP-AD with DIAG structure for additive + IDEN for dominance) and a MET model (i.e. ABLUP-AD and GBLUP-AD with CS/FAMK for additive + IDEN for dominance) were used. Based on the model comparison, CS were used for additive effects of Pilodyn, velocity, and MOE and FAMK were used for additive effects of tree height. In the MET models, additive effect *a/*$a_{1}$ in all equations [1–5] could be described as a=m+me, where *m* is the additive main marker/genetic effect (M), and *me* is the additive main marker-by-environment effect. Therefore, with CS and FAMK structures, the main marker effect (M), M + marker-by-environment interaction effect (A), and A + dominance effect (AD) from the GBLUP-AD and ABLUP-AD models could be estimated. In the CS model, m is the main term for markers and me is an interaction term for the markers and trials. All trials have the same marker variance and all pairs of trials have the same marker covariance, so that the var(a)= var(m)+var(me). A FAMK model is equivalent to a factor analytic model with (K+1) factors, where the first set of loadings are constrained to equal. $Var(a)=\;var(m)+\Lambda \Lambda^{T}+\Psi$, where Λ is a matrix of loadings and *Ψ* is a diagonal matrix with diagonal elements referred to as specific variances. In two-trial analyses, K=0, then var(a)= var(m)+ Ψ, which is equivalent to the CS+DIAG model (Table 1 and Oakey et al. (2016))

### Model Comparison

To compare the relative quality of the goodness-of-fit of the different models, the Akaike Information Criterion (AIC) and the fitted line plot (graph of predicted $\hat{y}$ vs. adjusted y values) were used for the linear mixed-effects models (LMM) for all traits, while the standard error of the predictions (SEPs) of the trait BVs was used to assess the precision of the BVs.

### Cross-validation

A 10-fold cross-validation scenario with 10 replications was used to assess accuracy and prediction ability (PA).

### Expected Performance of Genomic Selection

The expected performance of GS compared to standard phenotypic selection (PS) was evaluated only for the GBLUP-AD model by calculating the response to genomic selection (RGS) as a percentage of the population average as follows:

$$\begin{array}{l} RGS\left(\;\%\;\right)=\frac{\bar{EGV_{Gs}}-\bar{EGV_{o}}}{\bar{EGV_{o}}}\times 100 \end{array}$$

where $\bar{EGV_{Gs}}$ is the average of expected genetic values estimated from the ABLUP-AD model (equation [2]) for the selected portion of the population based on 1) main marker effects (M), 2) M + marker-by-environment interaction effects (A), and 3) A + dominance effects (AD) for site 1/site 2 estimated from GBLUP-AD model (equation [4]), respectively, and $\bar{EGV_{o}}$ is the population average (Resende et al. 2017). Response to phenotypic selection (RPS) as a percentage of the population average is as follows:

$$\begin{array}{l} RPS\left(\%\right)=\frac{\bar{EGV_{As}}-\bar{EGV_{o}}}{\bar{EGV_{o}}}\times 100 \end{array}$$

where $\bar{EGV_{As}}$ is the average of expected genetic values estimated from the ABLUP-AD model (equation [2]) for the selected portion of the population based on AD effects from the ABLUP-AD model. For different traits, ABLUP-AD and GBLUP-AD models with the best-fitting variance–covariance structures for additive and dominance variances were used (Table 2), except for Pilodyn data with CS for additive effects in order to permit comparison with ABLUP-AD results. The main advantage of using GS is that it permits a shorter breeding cycle. Thus, here we used RGS (%)/year and RPS (%)/year to compare the expected performances of GS and PS. In the Swedish Norway spruce breeding program, the traditional breeding cycle is at least 25 years long. If GS could be used as at a very early selection stage, the breeding cycle could be reduced to ca. 12.5 years (Chen et al. 2018).

**Table 2.** Summary of 5 models (2 ABLUP and 3 GBLUP models) with various variance and covariance structures fitted to the full data set for tree height, Pilodyn, velocity, and MOE

Trait	Model	Variance structure			Log-likelihood	AIC	No.
		Additive	Dominance	Epistasis			
Height	ABLUP-A	FAMK			−6873.47	13760.95	7
	**ABLUP-AD**	**FAMK**	**DIAG**		**−6868.92**	**13755.85**	**9**
	GBLUP-A	FAMK			−6874.05	13762.10	7
	**GBLUP-AD**	**FAMK**	**IDEN**		**−6870.21**	**13756.42**	**8**
	GBLUP-ADE	FAMK	IDEN	IDEN-G3*	−6870.21	13762.42	11
Pilodyn	**ABLUP-A**	**CS**			**−1727.77**	**3467.55**	**6**
	ABLUP-AD	CS	IDEN		−1727.77	3469.55	7
	**GBLUP-A**	**IDEN**			**−1737.44**	**3484.88**	**5**
	GBLUP-AD	IDEN	DIAG		−1735.87	3485.74	7
	GBLUP-ADE	IDEN	IDEN	IDEN-G3*	−1736.77	3493.54	10
Velocity	ABLUP-A	CS			1192.66	−2373.33	6
	**ABLUP-AD**	**CS**	**IDEN**		**1194.59**	**−2375.19**	**7**
	GBLUP-A	CS			1183.37	−2354.73	6
	**GBLUP-AD**	**CS**	**IDEN**		**1184.63**	**−2355.26**	**7**
	GBLUP-ADE	CS	IDEN	IDEN-G3*	1184.66	−2349.32	10
MOE	**ABLUP-A**	**CS**			**−2347.46**	**4706.92**	**6**
	ABLUP-AD	CS	IDEN		−2347.46	4708.92	7
	**GBLUP-A**	**CS**			**−2357.84**	**4727.67**	**6**
	GBLUP-AD	CS	IDEN		−2357.84	4729.67	7
	GBLUP-ADE	CS	IDEN	IDEN-G3*	−2357.84	4735.67	10

Variance and covariance structures: IDEN, identity; DIAG, diagonal; CS, compound symmetry; FAMK, factor analytic with the main marker/genetic term and k factors. * G3 represents GBLUP-ADE model including 3 first order epistatic effects (the random additive-by-additive epistatic effects, additive-by-dominance epistatic effects, and dominance-by-dominance epistatic effects). No. is the number of variance parameters. Bold means the best model in GBLUP or ABLUP.

## Results

### Genetic Variance Components and Heritability Estimates

**Figure 1.**
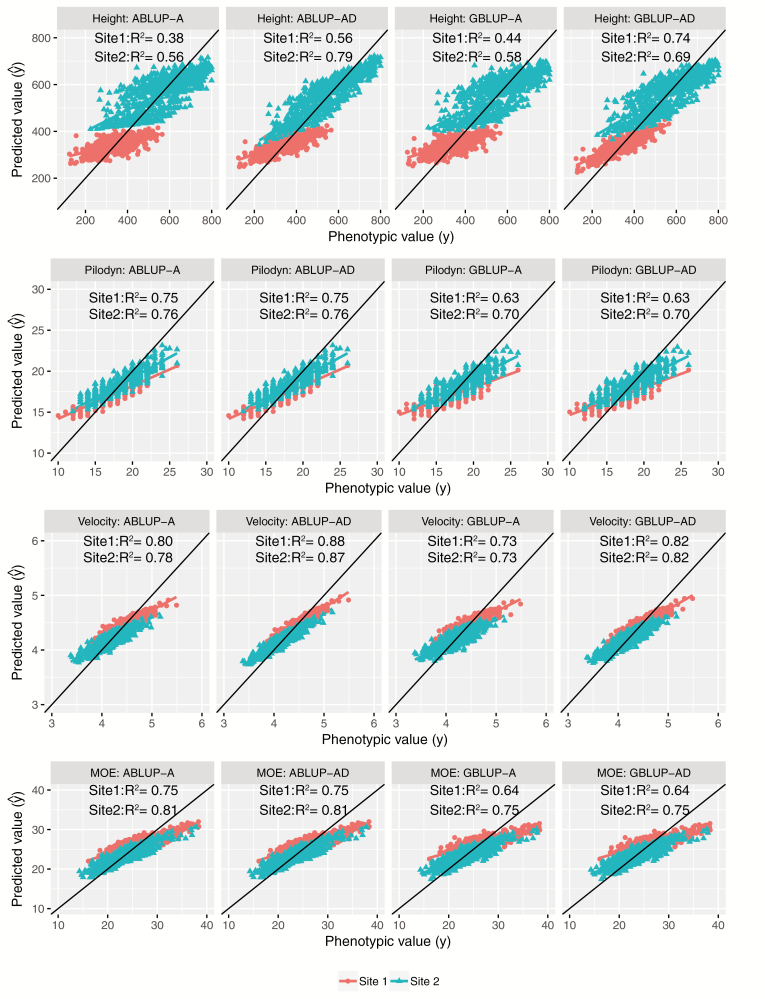
Model comparisons using the fitted line plots (represented by the graph of predicted values $\hat{y}$ vs observed values *y*) for tree height, Pilodyn, velocity, and MOE.

**Figure 2.**
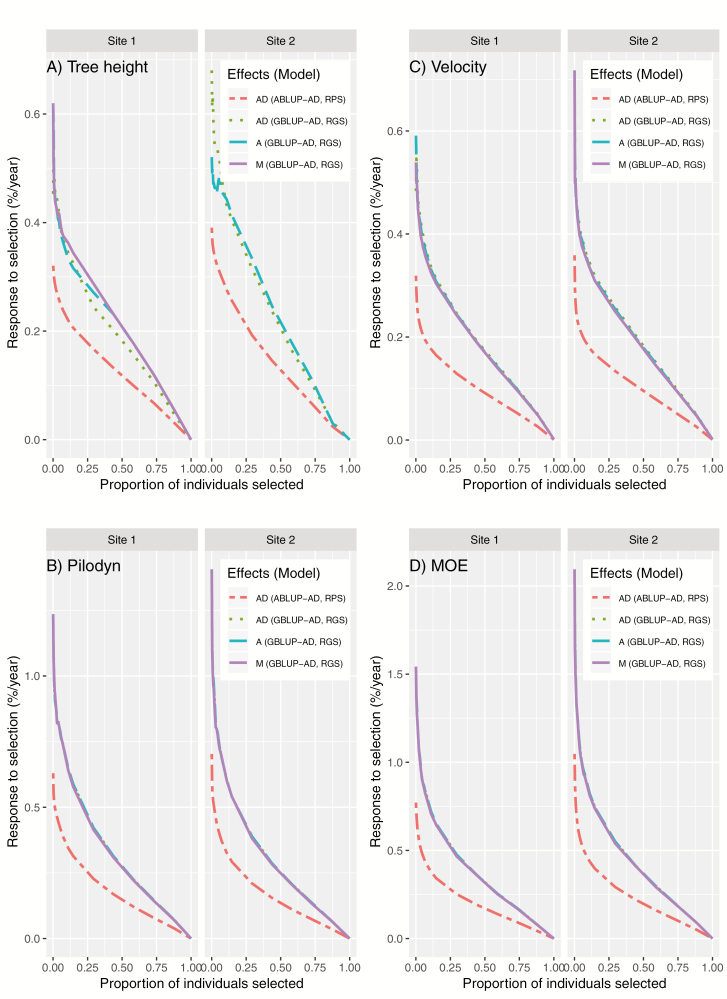
Response to genomic selection (RGS), including three different selection scenarios based on 1) only main marker effects (M), 2) main marker effects plus genotype-by-environment interaction effects (A), and 3) A + dominance (AD) from GBLUP-AD for A) tree height, B) Pilodyn, C) velocity, and D) MOE, expressed as a percentage gain of the average population mean per year, compared with response to phenotypic selection (RPS) also including dominance effects (ABLUP-AD) calculated for different proportions of individuals selected by GS.

The 6 variance and covariance structures examined for the additive, dominance, and epistatic effects are presented in Table 1. The log-likelihood, Akaike Information Criterion (AIC), and Bayesian Information Criterion (BIC) for the 5 models (ABLUP-A, ABLUP-AD, GBLUP-A, GBLUP-AD, and GBLUP-ADE) under various variance structures are shown in the Supplementary Material, Table S1. The models with the best fitted variance–covariance structures under ABLUP and GBLUP for additive variance only, additive plus dominance variance or additive plus dominance and epistasis (e.g. ABLUP-A, ABLUP-AD, GBLUP-A, GBLUP-AD, and GBLUP-ADE) are listed in Table 2. These were used to estimate the variance components (Table 3–5, Figure 1–2, except for Pilodyn with CS for additive effects and IDEN for dominance effects from GBLUP-A, GBLUP-AD, and GBLUP-ADE models). These models were included because we wanted to use the same variance–covariance structure to compare with the results from ABLUP-A and ABLUP-AD models for Pilodyn data (Table 2).

**Table 3.** Estimates of variance components (VC), their standard errors (SE) and the variance proportion of each site for tree height and velocity from the 5 genetic models fitted (ABLUP-A, ABLUP-AD, GBLUP-A, GBLUP-AD, and GBLUP-ADE)

Trait	VC	ABLUP-A		ABLUP-AD		GBLUP-A		GBLUP-AD		GBLUP-ADE	
		Value (SE)	%	Value (SE)	%	Value (SE)	%	Value (SE)	%	Value (SE)	%
Height	$\sigma_{b_{1}}^{2}$	815.5 (330.2)	12.1	804.0 (326.8)	11.9	772.4 (317.8)	11.5	703.1 (296.7)	10.4	703.1 (296.7)	10.4
	$\;\sigma_{b_{2}}^{2}$	1962.0 (655.8)	15.6	1863.3 (627.4)	14.9	1916.2 (643.0)	15.3	1918.6 (643.8)	15.3	1918.6 (643.8)	15.3
	$\;\sigma_{a_{1}}^{2}$	690.8 (368.4)	10.2	606.7 (392.1)	9.0	902.2 (395.4)	13.4	778.0 (407.2)	11.5	778.0 (407.2)	11.5
	$\sigma_{a_{12}}^{2}$	565.9 (374.6)		571.2 (368.5)		573.1 (406.4)		463.4 (409.6)		463.4 (409.6)	
	$\;\sigma_{a_{2}}^{2}$	2007.1 (717.5)	16.0	1371.5 (741.0)	11.0	2140.6 (736.4)	17.1	1858.7 (724.3)	14.8	1858.7 (724.3)	14.8
	$\;\sigma_{d_{1}}^{2}$			572.2 (925.7)	8.5			1224.1 (566.7)	18.1	1224.1 (566.7)	18.1
	$\sigma_{d_{2}}^{2}$			2881.9 (1443.9)	23.1			1224.1 (566.7)	9.8	1224.1 (566.7)	9.8
	$\sigma_{aa}^{2}$									0.00 (0.00)	0.0
	$\sigma_{ad}^{2}$									0.00 (0.00)	0.0
	$\sigma_{dd}^{2}$									0.00 (0.00)	0.0
	$\sigma_{e_{1}}^{2}$	5260.5 (421.9)	77.7	4777.6 (862.6)	70.7	5064.1 (416.8)	75.1	4053.3 (572.4)	60.0	4053.28 (572.5)	60.0
	$\sigma_{e_{2}}^{2}$	8604.5 (640.7)	68.4	6353.1 (1208.3)	50.9	8461.8 (679.1)	67.6	7523.9 (770.0)	60.1	7523.86 (770.1)	60.1
	$h_{1}^{2}$	0.12 (0.06)		0.10 (0.06)		0.15 (0.06)		0.13 (0.06)		0.13 (0.06)	
	$h_{2}^{2}$	0.19 (0.06)		0.14 (0.07)		0.20 (0.06)		0.18 (0.06)		0.18 (0.06)	
	$H_{1}^{2}$			0.20 (0.14)				0.33 (0.09)		0.33 (0.09)	
	$H_{2}^{2}$			0.40 (0.12)				0.29 (0.07)		0.30 (0.07)	
Velocity	$\sigma_{b_{1}}^{2}$	0.0018 (0.0013)	2.4	0.0019 (0.1355)	2.4	0.0013 (0.0011)	1.8	0.0014 (0.0011)	2.0	0.0014 (0.0011)	2.0
	$\;\sigma_{b_{2}}^{2}$	0.0036 (0.0018)	4.6	0.0034 (0.2356)	4.1	0.0033 (0.0017)	4.4	0.0034 (0.0017)	4.5	0.0034 (0.0017)	4.5
	$\;\sigma_{a_{1}}^{2}$	0.0365 (0.0087)	48.1	0.0343 (0.0087)	42.0	0.0305 (0.0051)	42.4	0.0290 (0.0052)	40.3	0.0282 (0.0065)	39.1
	$\sigma_{a_{12}}^{2}$	0.0320 (0.0086)		0.0293 (0.0087)		0.0241 (0.0051)		0.0224 (0.0052)		0.0215 (0.0066)	
	$\;\sigma_{a_{2}}^{2}$	0.0365 (0.0087)	46.8	0.0343 (0.0087)	40.9	0.0305 (0.0051)	40.3	0.0290 (0.0052)	38.3	0.0282 (0.0065)	37.2
	$\;\sigma_{d_{1}}^{2}$			0.0081 (0.0051)	9.9			0.0067 (0.0046)	9.3	0.0051 (0.0071)	7.1
	$\sigma_{d_{2}}^{2}$			0.0081 (0.0051)	9.6			0.0067 (0.0046)	8.8	0.0051 (0.0071)	6.8
	$\sigma_{aa}^{2}$									0.0030 (0.0152)	4.2/4.0
	$\sigma_{ad}^{2}$									0 (0)	0
	$\sigma_{dd}^{2}$									0.0005 (0.0108)	0.7/0.7
	$\sigma_{e_{1}}^{2}$	0.0376 (0.0057)	49.5	0.0373 (0.0057)	45.7	0.0402 (0.0042)	55.8	0.0349 (0.0052)	48.4	0.0336 (0.0077)	46.7
	$\sigma_{e_{2}}^{2}$	0.0379 (0.0053)	48.6	0.0381 (0.0053)	45.4	0.0418 (0.0040)	55.3	0.0367 (0.0050)	48.4	0.0355 (0.0079)	48.1
	$h_{1}^{2}$	0.43 (0.10)		0.40 (0.10)		0.34 (0.06)		0.32 (0.07)		0.31 (0.09)	
	$h_{2}^{2}$	0.43 (0.09)		0.39 (0.10)		0.33 (0.06)		0.31 (0.07)		0.30 (0.09)	
	$H_{1}^{2}$			0.51 (0.10)				0.41 (0.08)		0.43 (0.11)	
	$H_{2}^{2}$			0.50 (0.10)				0.40 (0.08)		0.42 (0.11)	

Note: $\;\sigma_{b_{1}}^{2}$and $\sigma_{b_{2}}^{2}$. are the block variance for site 1 and site 2. $\sigma_{a1}^{2}$$\sigma_{a2}^{2}$, and $\sigma_{a12}$ are the additive variances for site 1, site 2, and additive covariance between site 1 and site 2, respectively. $\sigma_{d1}^{2}$$\sigma_{d2}^{2}$, and $\sigma_{d12}$ are the dominance variances for site 1, site 2, and dominance covariance between site 1 and site 2. $\;\sigma_{aa}^{2}$, $\sigma_{ad}^{2}$, and $\sigma_{dd}^{2}$ are the additive × additive epistatic variance, additive × dominance epistatic variance, and dominance × dominance epistatic variances, respectively. $\Sigma_{e1}^{2}$ and $\Sigma_{e2}^{2}$ are the residual variances for site 1 and site 2, respectively. $h_{1}^{2}$ and $h_{2}^{2}$ are the narrow-sense heritability for site 1 and site 2, respectively. $H_{1}^{2}$ and $H_{2}^{2}$ are the broad-sense heritability for site 1 and site 2, respectively.

M×E effects for the additive or nonadditive effects were considered significant if the AIC values in MET analyses (e.g. under CS and FAMK variance structures) were smaller than the corresponding AIC values in single site (ST) analyses (e.g. under IDEN or DIAG variance structure only) for the same trait or if the Log-likelihood Ratio test (LRT) was significant. All models with CS for additive genetic effects were found performing best, except for the model with FAMK for tree height additive genetic effects (Table 2). Based on this criterion, all 4 traits showed significant additive M×E effects, except for the Pilodyn trait under GBLUP models. However, additive-by-environment variance in site 1 from ABLUP-AD with FAMK was not significant (Table 3, 606.7) when assessed on the AIC. For the dominance effect, however, only the tree height with IDEN and velocity with DIAG structure had significant effects: therefore, there was no significant M×E for a dominance effect of any trait. For epistasis, there was no significant effect on any trait.

Estimates of variance components, their standard errors (SE), and the variance proportion of each site for tree height and velocity from the 5 genetic models fitted (ABLUP-A, ABLUP-AD, GBLUP-A, GBLUP-AD, and GBLUP-ADE) are shown in Table 3 and the results of Pilodyn and MOE are shown in Table S2. Block variance components ($\sigma_{b}^{2}$) for each site were almost consistent across the 5 models for all traits (Table 3 and Table S2). For example, $\sigma_{b}^{2}$ for tree height accounted for 10.4%−12.9% and 14.9%−15.6% for sites 1 and 2, respectively. For tree height, the main difference between the ABLUP-A and GBLUP-A models was the substantial increase of the additive variance ($\sigma_{a}^{2}$) (Table 3), in contrast to results for wood quality traits. For example, tree height additive variances $\sigma_{a}^{2}$s estimated from GBLUP-A were 130.6% and 106.7% of the ABLUP-A $\sigma_{a}^{2}$s at site 1 and site 2, respectively. However, Pilodyn and velocity additive variances $\sigma_{a}^{2}$s estimated from GBLUP-A averaged 77.8% and 83.6% of the ABLUP-A $\sigma_{a}^{2}$s for both sites. The tree heights $\sigma_{a}^{2}$s estimated from GBLUP-AD were also larger than those from ABLUP-AD for both sites. In contrast, wood quality traits $\sigma_{a}^{2}$s estimated from GBLUP-AD were also smaller than those from ABLUP-AD for both sites. For tree height and velocity, the main differences between ABLUP-A and ABLUP-AD and between GBLUP-A and GBLUP-AD were the substantial decrease in $\sigma_{a}^{2}\;$(Table 3). Pilodyn and MOE had the same $\sigma_{a}^{2}$s for the ABLUP-A and ABLUP-AD and also for GBLUP-A and GBLUP-AD because dominance variances ($\sigma_{d}^{2}$s) were zero for both traits (Table S3). For example, tree height $\sigma_{a}^{2}$s estimated from ABLUP-AD were 87.8% and 68.3% of the $\sigma_{a}^{2}$s estimated from ABLUP-A at site 1 and site 2, respectively.

In the ABLUP-AD model, tree height and velocity dominances showed significant effects based on the AIC (Tables 2 and 3). For example, tree height dominance effects accounted for 8.5% and 23.1% of the phenotypic variation for site 1 and site 2, respectively. In the GBLUP-AD model, tree height dominance effects accounted for 18.1% and 9.8% of the phenotypic variation for site 1 and site 2, respectively. However, based on the AIC, the dominance variance of 572.2 at site 1 was not significant. In the GBLUP-ADE models, first-order epistatic effects were all zero for all the 4 traits, except for velocity with nonsignificant additive × additive effects (4.2%) and dominance × dominance effects (0.7%) (Table 3).

Estimates of tree height and velocity narrow-sense heritability from ABLUP-A or GBLUP-A models were larger than those from ABLUP-AD or GBLUP-AD. For example, tree height narrow-sense heritability of 0.12 from ABLUP-A was larger than 0.10 from ABLUP-AD at site 1. Broad-sense heritability estimates were substantially larger than narrow-sense heritability estimates from both ABLUP-AD and GBLUP-AD at both sites for tree height and velocity. For example, tree height broad-sense heritability estimates were 253.8% and 166.7% of the narrow-sense heritability estimates from the GBLUP-AD model at site 1 and site 2, respectively. For tree height, Pilodyn and MOE, GBLUP-ADE produced exactly the same results as GBLUP-AD (Table 3 and Supplementary Material, Table S2) because of the lack of epistasis. In this study, only velocity showed nonsignificant and nonzero epistatic effects. Moreover, broad-sense heritability estimates from the GBLUP-ADE models were slightly higher than those from GBLUP-AD (0.43 vs. 0.41 for site 1 and 0.42 vs. 0.40 for site 2).

### Comparison of Models

We used 2 methods for model comparison, namely AIC (Supplementary Material, Table S1 and Table 2) and the fitted line plots (represented by the graph of predicted values $\hat{y}$ vs. observed values *y*) (Figure 1). The fitted line plot comparisons based on R^2^ reflected the goodness-of-fit. For tree height and velocity, R^2^ increased from GBLUP-A to GBLUP-AD (Tree height: 0.38 vs. 0.56 in site 1 and 0.56 vs 0.79 in site 2; velocity: 0.80 vs. 0.88 in site 1 and 0.78 vs. 0.87 in site 2) and from ABLUP-A to ABLUP-AD for both sites (Tree height: 0.44 vs. 0.74 in site 1 and 0.58 vs 0.69 in site 2; velocity: 0.73 vs. 0.82 in site 1 and 0.73 vs. 0.82 in site 2). For Pilodyn and MOE, *R*^2^ was the same from GBLUP-A to GBLUP-AD and from ABLUP-A to ABLUP-AD, which was consistent with the zero estimates of dominance variances for both traits (Supplementary Material, Table S2). The difference of *R*^2^ for tree heights between site 1 and site 2 was much larger than that of wood quality traits for all models.

A comparison of BVs’ precision using the standard errors for the predictions (SEPs) between different models (GBLUP-AD vs. GBLUP-A, GBLUP-AD vs. ABLUP-AD, GBLUP-AD vs. ABLUP-A, GBLUP-A vs. ABLUP-AD, GBLUP-A vs. ABLUP-A, and ABLUP-AD vs. ABLUP-A) is shown in Supplementary Material, Figure S1 for all traits. For tree height, the SEPs of 21-year-old Norway spruce breeding values between ABLUP-AD and ABLUP-A showed similar values. GBLUP-AD for tree height had much lower SEPs than that of GBLUP-A, but not for wood quality traits. GBLUP-AD for all traits had much lower SEPs values than that from ABLUP-AD for most SEPs values. ABLUP-AD for all traits had almost the same SEPs as ABLUP-A, even for tree height. For all traits, GBLUP-AD and GBLUP-A had more and lower SEPs than those from ABLUP-AD and ABLUP-A, except the GBLUP-A for tree height had more and larger SEPs than those from ABLUP-A and ABLUP-AD.

### Cross-Validation of the Models

A random selection of 10% of the population was used as a validation set. To test the ranking difference of estimated breeding values between 5 models, Spearman’s rank correlations were used (Table 4). Spearman’s rank correlations between breeding values estimated by pedigree-based models (ABLUP-A and ABLUP-AD) and between breeding values estimated by genomic-based models (GBLUP-A and GBLUP-AD) in cross-validation were higher than between pedigree-based and genomic-based models (Table 4). For example, Spearman’s rank correlations between breeding values estimated by pedigree-based and genomic-based models for tree height were 0.884. Spearman’s rank correlations between breeding values estimated by within pedigree-based models or genomic-based models were almost the same. For example, Spearman’s rank correlation between breeding values estimated by ABLUP-A and ABLUP-AD for tree height were 1.00.

**Table 4.** Coefficients of Spearman’s rank correlations between breeding values estimated by ABLUP-A, ABLUP-AD, GBLUP-A, and GBLUP-AD in cross-validation for tree height, Pilodyn, velocity, and MOE

Trait	ABLUP-A	ABLUP-AD	GBLUP-A	GBLUP-AD
Height				
ABLUP-A		0.997	0.877	0.876
ABLUP_AD	0.998		0.873	0.873
GBLUP-A	0.884	0.878		0.995
GBLUP-AD	0.879	0.875	1	
Pilodyn				
ABLUP_A		1	0.818	0.819
ABLUP-AD	1		0.818	0.819
GBLUP-A	0.819	0.819		1
GBLUP-AD	0.820	0.820	1	
Velocity				
ABLUP_A		0.998	0.868	0.869
ABLUP-AD	0.998		0.868	0.868
GBLUP-A	0.869	0.869	1	0.999
GBLUP-AD	0.869	0.869	0.999	1
MOE				
ABLUP_A		1	0.837	0.837
ABLUP-AD	1		0.837	0.837
GBLUP-A	0.837	0.837		1
GBLUP-AD	0.837	0.837	1	

ABLUP-A, ABLUP-AD, GBLUP-A, and GBLUP-AD with the best variance structure are based on AIC in Table 2.

The cross-validation focused on comparing the predictive ability (PA) between GBLUP-AD and ABLUP-AD models and between MET and single-trial (ST) models for all traits; results are shown in Table 5. We examined only the models with either CS or FAMK for additive effects and either CS or IDEN for dominance effects in the MET analysis. For a single trial (ST) analysis, the models with DIAG for additive and IDEN or DIAG for dominance effects based on Table 2 were used. Using the same site data as a training set and a validation set showed higher PA. Tree height PA from the ST analysis at site 2 was higher than that at site 1 for additive effects (A) from GBLUP-AD models (comparisons: 1 and 3, 0.25 vs. 0.24, Table 5) and ABLUP-AD models (comparisons: 1 and 3, 0.26 vs. 0.21, Table 5). The models with additive and dominance effects (AD) showed results similar to those of the models with an additive effect only (A) for tree height. If 1 site was used to build the model and predict the breeding values (A) and genotype values (AD) for the second site, then predicting for site 2 using the models from site 1 had a higher PA than the opposite for both GBLUP-AD (comparisons: 2 and 4, 0.09 vs. 0.07, Table 5) and ABLUP-AD (comparisons: 2 and 4, 0.13 vs. 0.09, Table 5). Ly et al. (2013) suggested that G×E, which cannot be estimated for a single trial, reduced the ability to make predictions. Our results proved that the site 2 tree height might have a higher environmental component than that observed in site 1, making the prediction of the BVs (additive) or genetic values (GVs: additive and dominance) less accurate. PA of Pilodyn did not change, or only slightly changed, using site 1 model for site 2 and vice versa. This happened because there is almost no G×E in Pilodyn measurements.

**Table 5.** Predictive abilities (PA) based on main marker effects (M), M + marker-by-environment interaction effects (A) and A + dominance effects (AD) from GBLUP-AD and ABLUP-AD models for tree height, Pilodyn, velocity, and MOE in the single trial (ST) and multi-environment trial (MET) model analysis in cross-validation

Trait	Comparison	Type	Structure^a^	Training	Validation	GBLUP-AD			ABLUP-AD		
						M	A	AD	M	A	AD
Height	1	ST	DIAG+IDEN	Site 1	Site1	N/A	0.24 (0.04)	0.26 (0.03)	N/A	0.21 (0.04)	0.20 (0.04)
	2	ST	DIAG+IDEN	Site 1	Site2	N/A	0.09 (0.03)	0.16 (0.03)	N/A	0.13 (0.03)	0.12 (0.03)
	3	ST	DIAG+IDEN	Site 2	Site2	N/A	0.25 (0.03)	0.27 (0.03)	N/A	0.26 (0.04)	0.29 (0.04)
	4	ST	DIAG+IDEN	Site 2	Site1	N/A	0.07 (0.04)	0.12 (0.04)	N/A	0.09 (0.03)	0.08 (0.03)
	5	MET	FAMK+IDEN	Site 1	Site 1	0.22 (0.04)	0.23 (0.04)	0.26 (0.03)	0.19 (0.03)	0.19 (0.03)	0.20 (0.03)
	6	MET	FAMK+IDEN	site 2	Site 2	0.22 (0.04)	0.25 (0.03)	0.27 (0.03)	0.21 (0.03)	0.24 (0.03)	0.29 (0.04)
Pilodyn	1	ST	DIAG+IDEN	Site 1	Site 1	N/A	0.26 (0.05)	0.27 (0.05)	N/A	0.30 (0.05)	0.30 (0.05)
	2	ST	DIAG+IDEN	Site 1	Site 2	N/A	0.23 (0.04)	0.23 (0.04)	N/A	0.24 (0.03)	0.25 (0.03)
	3	ST	DIAG+IDEN	Site 2	Site 2	N/A	0.23 (0.03)	0.31 (0.03)	N/A	0.34 (0.02)	0.33 (0.02)
	4	ST	DIAG+IDEN	Site 2	Site 1	N/A	0.23 (0.03)	0.23 (0.03)	N/A	0.23 (0.03)	0.24 (0.03)
	5	MET	CS+ IDEN	Site 1	Site 1	0.30 (0.04)	0.30 (0.04)	0.30 (0.04)	0.32 (0.03)	0.33 (0.04)	0.33 (0.04)
	6	MET	CS+IDEN	Site 2	Site 2	0.32 (0.03)	0.32 (0.03) (0.03)	0.32 (0.03)	0.34 (0.02)	0.35 (0.02)	0.35 (0.02)
Velocity	1	ST	DIAG+IDEN	Site 1	Site 1	N/A	0.44 (0.04)	0.45 (0.04)	N/A	0.40(0.04)	0.42 (0.04)
	2	ST	DIAG+IDEN	Site 1	Site 2	N/A	0.32 (0.03)	0.33 (0.02)	N/A	0.35 (0.03)	0.36 (0.03)
	3	ST	DIAG+IDEN	Site 2	Site 2	N/A	0.38 (0.02)	0.39 (0.02)	N/A	0.40 (0.04)	0.41 (0.04)
	4	ST	DIAG+IDEN	Site 2	Site 1	N/A	0.34 (0.06)	0.35 (0.06)	N/A	0.36 (0.04)	0.38 (0.04)
	5	MET	CS+IDEN	Site 1	Site 1	0.45 (0.05)	0.46 (0.04)	0.46 (0.04)	0.42 (0.04)	0.43 (0.04)	0.43 (0.04)
	6	MET	CS+IDEN	Site 2	Site 2	0.39 (0.03)	0.39 (0.03)	0.39 (0.03)	0.42 (0.04)	0.43 (0.04)	0.43 (0.04)
MOE	1	ST	DIAG+IDEN	Site 1	Site 1	N/A	0.33 (0.03)	0.33 (0.03)	N/A	0.34 (0.03)	0.35 (0.03)
	2	ST	DIAG+IDEN	Site 1	Site 2	N/A	0.28 (0.04)	0.28 (0.04)	N/A	0.31 (0.04)	0.32 (0.04)
	3	ST	DIAG+IDEN	Site 2	Site 2	N/A	0.33 (0.03)	0.33 (0.04)	N/A	0.36 (0.04)	0.36 (0.04)
	4	ST	DIAG+IDEN	Site 2	Site 1	N/A	0.30 (0.04)	0.30 (0.04)	N/A	0.32 (0.04)	0.32 (0.04)
	5	MET	CS+IDEN	Site 1	Site 1	0.37 (0.04)	0.37 (0.04)	0.37 (0.04)	0.38 (0.03)	0.39 (0.03)	0.39 (0.03)
	6	MET	CS+IDEN	Site 2	Site 2	0.35 (0.04)	0.35 (0.04)	0.35 (0.04)	0.38 (0.04)	0.38 (0.04)	0.38 (0.04)

Standard errors are in parentheses.

^a^Including additive structure plus dominance structure.

Generally, PA was higher in the MET analysis than that in ST analysis for all traits, except for tree height (Table 5). For Pilodyn, velocity, and MOE, PAs in MET analyses based on A and AD effects were higher than those from single site (ST) analyses (comparisons 1 and 5, comparisons 2 and 6, Table 5). For example, PAs for Pilodyn based on A from GBLUP-AD showed an increase of 15.4% (comparisons 1 and 5, 0.26 vs. 0.30, Table 5) and 39.1% (comparisons 3 and 6, 0.23 vs. 0.32, Table 5) in sites 1 and 2, respectively.

Finally, we studied the additive M×E effects on the genomic-based estimated breeding values (GEBVs). There was a reduction in tree height PA if M×E was not included in calculating the GEBVs for site 2 (comparison 6: 0.25 vs. 0.22, Table 5), and for site 1 (comparison 5: 0.23 vs. 0.22, Table 5). Including tree height dominance in models in site 2, PA increased 8% from 0.25 to 0.27 and 20.8% from 0.24 to 0.29 for GBLUP-AD and ABLUP-AD models, respectively (Table 5). Including tree height dominance in models in site 1, PA increased 13.0% from 0.23 to 0.26 and 5.3% from 0.19 to 0.20 for GBLUP-AD and ABLUP-AD models, respectively. For Pilodyn, velocity, and MOE, PA including dominance in MET analysis was not increased, even for velocity with a significant dominance variance based on AIC.

Predictive ability (PA) for all traits from GBLUP-ADE is not shown in Table 5 because their variance components were zero, except for velocity. PA for velocity from the GBLUP-ADE model was the same as the result from GBLUP-AD.

### Expected Response to Genomic Selection (GS)

We compared the generation time of GS (ca. 12.5 years) with the generation time of the phenotypic selection (ca. 25 years), as in the traditional breeding program in Northern Sweden (Chen et al. 2018). A conservative response of genomic selection per year (RGS%/year) was calculated to compare with the response of phenotypic selection per year (RPS%/year) for variable proportions of individuals selected by GS. We compared RGS per year with RPS per year for all traits for variable proportions of individuals selected by GS (Figure 2). The results showed that RGS per year provided much larger values than RPS per year for 3 genomic selection scenarios, including selection based on 1) main marker effects (M), 2) M plus M×E effects (A), and 3) A plus dominance effects (AD) from GBLUP-AD model for both sites (Figure 2). However, RGS per year for different scenarios in both sites showed slight differences only for tree height, not for wood quality traits. RGS per year for tree height based on A and AD was substantially higher than that based on M in site 2 (Figure 2). However, in site 1, RGS per year for tree height based on A and AD was slightly better than that based on M and showed only at a low selection proportion. In the traditional Swedish breeding program, 50 individuals were selected for each breeding population. In Supplementary Material, Figure S2, the top 50 individuals selected without considering relationships for selection based on M, A, and AD for all traits were scaled to the total expected genetic value (EGV) ranking of all individuals in sites 1 and 2. RGS per year based on M, A, and AD for GBLUP-AD, and RPS per year based on an AD for ABLUP-AD, are shown in Supplementary Material, Table S3. For tree height, RGS per year based on AD in site 2 was 0.54 (%)/year, which was substantially higher than 0.45 and 0.46 (%)/year based on A and M, respectively. RGS per year based on AD in site 1 was 0.43 (%)/year, which was slightly higher than 0.41 and 0.41 (%)/year based on A and M, respectively. For wood quality traits, RGS per year based on M, A, and AD were almost the same (Figure 2), but they slightly increased when such effects were significant (Supplementary Material, Table S3). If the top 50 velocity individuals based on genomic-based expected genetic values were selected, RGS per year from GBLUP-AD were 78.9%, 86.9%, and 91.3% in site 1, and 80.8%, 82.9%, and 88.2% in site 2, higher than RPS (%)/year based on M, A, and AD effects, respectively. RGS per year from GBLUP-AD for tree height, Pilodyn, and MOE were up to 68.9%, 91.3%, and 92.6%, respectively.

## Discussion

### Genetic Variance Components and Heritability Estimates

In the traditional Norway spruce breeding program, estimates of broad-sense heritability (*H*^2^) have previously been made in tests of clones selected from commercial nurseries and with an unknown family structure. For example, tree height *H*^2^ estimates vary from 0.12 to 0.40 for Norway spruce (Bentzer et al. 1989; Karlsson and Högberg 1998), but it is not possible to compare with narrow-sense heritability (*h*^2^), which requires a family structure. Using a traditional pedigree-based model, epistasis estimation, on the other hand, requires full-sib family structure plus the replication of genotypes in clonal trials. Existing high-throughput single nucleotide polymorphism (SNP) genotyping technology, such as SNP arrays, re-sequencing, or genotyping-by-sequencing (GBS), allows genotyping larger numbers of SNPs, and therefore is used to study dominance and epistasis in populations without pedigree delineation of full-sib family structure in both animals (Sun et al. 2013; Aliloo et al. 2016) and plants (Gamal El-Dien et al. 2018).

In our study, tree height *H*^2^ estimated from ABLUP-AD (0.20–0.40) was higher than *h*^2^ estimated from pedigree-based ABLUP-A and ABLUP-AD (0.10–0.19) (Table 3). In a previous study, it was however observed that the average *h*^2^ of 0.29 (0.02–1.09) based on 170 field tests with seedlings was higher than the average *H*^2^ of 0.18 (0.04–0.50) based on 123 field tests with clonal material (Kroon et al. 2011), indicating that a valid comparison of relative genetic control must use the datasets that come from the same trial with comparable pedigree (Wu 2018). The ratio of tree height *h*^2^/*H*^2^ varies from 0.35–0.50 ($\sigma_{D}^{2}/\sigma_{A}^{2}=2.10--0.94$) and from 0.39–0.60 ($\sigma_{D}^{2}/\sigma_{A}^{2}=1.60--0.67$) in ABLUP-AD and GBLUP-AD models, respectively. These figures are lower than 0.60–0.84 ($\sigma_{D}^{2}/\sigma_{A}^{2}=0.67--0.19$) from 3 Norway spruce progeny trials in the previous study (Kroon et al. 2011). The usual range of the ratio *h*^2^/*H*^2^ has been reported to vary from 0.18 to 0.84 ($\sigma_{D}^{2}/\sigma_{A}^{2}=4.56--0.19$) for tree traits (Wu 2018). It is also considered that significant dominance could be utilized in advanced Norway spruce breeding and deployment programs.

Our results show that the inclusion of dominance effects reduces estimates of *h*^2^ from GBLUP-AD and ABLUP-AD when dominance is not zero. For example, tree height *h*^2^ estimates decrease by 13%–26%, less than the substantial decrease (50%–70%) reported in hybrid *Eucalyptus* by Tan et al. (2018). The situation is expected from a theoretical standpoint as a substantial proportion of the nonadditive variance can be inseparable from additive variance (Falconer and Mackay 1996), that has been encountered in the several empirical studies (Gamal El-Dien et al. 2016, 2018; Tan et al. 2018).

### Comparison and Cross-Validation of Models

AIC values for the GBLUP models were not significantly higher than those based on pedigree relationship matrices, which is consistent with the results of Gamal El-Dien et al. (2016) in white spruce, but is in contrast with the results from hybrid *Eucalyptus* (Tan et al. 2018). In the latter, the significant improvement from GBLUP models based on AIC may result from a considerable number of uncorrected pedigrees including a labelling mistake. In our study, the SEPs of breeding values in GBLUP-A models for tree height were higher than that in pedigree-based ABLUP-A model (In Supplementary Material, Figure S1), which is inconsistent with the results of Gamal El-Dien et al. (2018). This seems reasonable in our study because the additive variance increases from ABLUP models to GBLUP models (Table 3). For wood quality traits, the SEPs of breeding values in GBLUP-A models are smaller than those in the pedigree-based ABLUP-A model. For all traits, most SEPs of breeding values in GBLUP-AD model are smaller than in GBLUP-A, ABLUP-AD, and ABLUP-A models, which indicates that GBLUP-AD could produce more accurate breeding values, even though the Spearman’s rank correlations between breeding values estimated by GBLUP-AD and GBLUP-A are similar.

### M×E for Genomic Selection in Multi-Environment Trials (METs)

Gamal EL-Dien et al. (2018) showed that interior spruce (*Picea glauca* x *engelmannii*) had substantially significant additive M×E and nonsignificant small dominance M×E terms for both height and wood density. In our study, significant but small M×E effects for all traits were found only in additive genetic effects, not for dominance. Gamal EL-Dien et al. (2018) did not use more comprehensive models to dissect M×E, but used compound symmetry variance–covariance structures (CS). To more accurately dissect M×E in multi-environment trials (METs), here we used 6 variance–covariance matrices (Table 1) to model additive and dominance effects in GS models for 4 traits. A similar approach was described by Burgueño et al. (2012), Oakey et al. (2016) and Ventorim Eerrao et al. (2017). Finally, we found that all 4 traits have significant additive M×E terms using CS for additive effects. For tree height, we also observed a better goodness-of-fit using FAMK in Supplementary Material, Table S1. Here we should note that site 1 has a nonsignificant additive M×E term for tree height that resulted in a negligible increase with the M×E term included in the GBLUP-AD model. Generally, MET analysis shows slightly higher PA than does ST analysis, except for tree height which has the same value. This may result from the nonsignificant additive covariance ($\sigma_{a_{12}}^{2}$) between 2 sites. In this study, only tree height and velocity had slight increases for PA, which also supports the previous study of Ly et al. (2013) that including the G×E term could improve the PA.

### Significant Dominance Effects Improve Predictive Ability

Recent studies have shown that maximum PA can be reached when the model is based on additive and nonadditive effects (Da et al. 2014; Muñoz et al. 2014; Aliloo et al. 2016; Tan et al. 2018). Ly et al. (2013) considered that only the additive component may produce a systematic underestimation of PA because only additive effects are predicted. Here, the GBLUP-AD model for tree height shows homogenous dominance variances in both sites (Table 3, identity matrix for dominance effect). However, the ABLUP-AD model shows a significant dominance variance in site 2 (23.1%) and nonsignificant dominance variance in site 1 (8.5%), indicating that the GBLUP-AD model has higher efficiency in separating the additive and dominance genetic variances because it could account for the Mendelian sampling within families for dominance.

It was found that including dominance could improve PA when a considerable dominance variance in animal (Aliloo et al. 2016; Esfandyari et al. 2016) and plant studies were observed (Wolfe et al. 2016; Tan et al. 2018;). In this study, the improvement of tree height and velocity PAs also agree with the previous observations. However, including significant dominance in this study may not improve the Spearman’s rank correlations between breeding values (Table 4).

A dominance effect has been used in several practical breeding programs, such as loblolly pine (McKeand et al. 2003), Sitka spruce (*Picea sitchensis*) (Thompson 2013), and eucalypts (Rezende et al. 2014). For instance, since 2000, the annual production of full-sib seedlings in loblolly pine increased to 63.2 million in 2013, with a total of over 325 million full-sib family seedlings planted over the last 14 years (Steve Mckeand 2014, personal communication). In Norway spruce, a dominance estimate was not widely included in the breeding program, but we are commonly using full-sib family material. Thus, it is important to estimate dominance effects in the Norway spruce breeding program as more and more individuals will be genotyped for selection and propagation.

### Epistasis Effect

The full model (GBLUP-ADE), which was extended to include 3 first-order interactions, shows almost the same results as GBLUP-AD for all 4 traits based on AIC (Table 2). This indicates the absence of 3 kinds of epistatic interactions even though additive × additive and dominance × dominance epistatic effects explained variations of 4.2% (4.0%) and 0.7% (0.2%) for velocity in site 1 (site2), respectively. However, in several other forest tree species, such as white spruce (Gamal El-Dien et al. 2016), loblolly pine (de Almeida Filho et al. 2016), eucalypt (Bouvet et al. 2016; Resende et al. 2017; Tan et al. 2018), and interior spruce (*P. glauca x engelmannii*) (Gamal El-Dien et al. 2018), significant epistatic effects have been reported for height or wood density. For instance, Gamal El-Dien et al. (2016) showed a significant additive × additive component and nonsignificant dominance for tree height, while Gamal El-Dien et al. (2018) showed a considerable dominance component (19.46% of total phenotypic variation) and no epistatic effect. For wood density in spruce, Gamal El-Dien et al. (2016, 2018) showed a significant additive × additive interaction that was absorbed from additive and residual variances. Tan et al. (2018) showed no epistasis for wood density. The above results agree with the suggestion by Tan et al. (2018) that the contributions of nonadditive effects, especially epistasis effects, are traits, populations, and species-specific, or even site-specific as in this study. However, including significant nonadditive effects could improve estimates of genetic parameters.

### Expected Response to Genomic Selection

The main advantages of GS are to shorten the length of the breeding cycle and reduce phenotypic evaluation costs in plant and animal breeding (Grattapaglia and Resende 2011; de los Campos et al. 2013). In Northern Sweden, the length of the breeding cycle of Norway spruce in GS could be ideally shortened from 25 years to 12.5 years (Chen et al. 2018) if we could complete flowering induction and controlled pollinations within 12.5 years. In our previous paper (Chen et al. 2018), we calculated the RGS per year for GS based on GBLUP-A using the same data set. Here we compared RPS with RGS per year for GS based on a GBLUP-AD model and calculated the response to selection per year for PS and GS. We used EGVs from an ABLUP-AD model as a benchmark for all traits. RGS per year is considerably higher than RPS per year for all traits (Figure 2). RGS per year for wood quality traits has greater gain than those for tree height when we select the top 50 individuals based on a M, A or AD effect, in contrast to the result reported by Resende et al. (2017) for *Eucalyptus*. Thus, GS based on genomic-based expected genetic values is ideal for solid-wood quality improvement in Norway spruce.

## Conclusions

This is the first paper to study M×E using a different covariance structure for the additive and nonadditive effects and dominance in GS for forestry trees species. We found that M×E and dominance effects could improve PA when they are appreciably large. In a GBLUP-AD model, M×E contributed 4.7% and 11.1% of tree height phenotypic variation for sites 1 and 2, respectively. Dominance contributed 18.1% and 9.8% of tree height phenotypic variation for sites 1 and 2, respectively. The higher PA of the GBLUP-AD model for tree height compared to ABLUP-A and GBLUP-A models suggests that dominance should be included in GS models for genetic evaluations in forestry to improve the predictive accuracy or estimates of genetic parameters. Advanced M×E models could improve PA and should be included in the model fitting. GBLUP-AD could be a more useful model in breeding and propagation when tree breeders want to use the dominance using full-sib family seedlings.

## Funding

Financial support was received from Formas (grant number 230-2014-427), the Swedish Foundation for Strategic Research (SSF, grant number RBP14-0040), and from the European Union’s Horizon 2020 research and innovation programme under grant agreement No 773383 (B4EST project).

## Supplementary Material

esz061_suppl_Supplementary_Materials

## Data Availability

The data is archived in the Dryad Data Repository https://doi.org/10.5061/dryad.pk0p2nghn.
