# Supplementary material for: Increased Prediction Ability in Norway Spruce Trials Using a Marker X Environment Interaction and Non-Additive Genomic Selection Model
Source: J Hered. 2019 Oct 20;110(7):830–43. doi: 10.1093/jhered/esz061 (PMC6916663; doi:10.1093/jhered/esz061)
Supplement: esz061_suppl_Supplementary_Materials [file esz061_suppl_supplementary_materials.docx]

**Table S1**. Summary of the five models (two ABLUP and three GBLUP models) with various variance and covariance structures fitted to the full data set for tree height, Pilodyn, velocity, and MOE

| Trait | Model |  | Variance and covariance structure | | |  | ST or MET | Log-likelihood | AIC | BIC | No. |
| --- | --- | --- | --- | --- | --- | --- | --- | --- | --- | --- | --- |
|  |  |  | Additive | Dominance | Epistasis |  |  |  |  |  |  |
| Tree height | ABLUP-A |  | IDEN |  |  |  | ST | -6879.0 | 13768.0 | 13794.11 | 5 |
|  | ABLUP-A |  | DIAG |  |  |  | ST | -6874.7 | 13761.4 | 13792.68 | 6 |
|  | ABLUP-A |  | CS |  |  |  | MET | -6875.14 | 13762.3 | 13793.61 | 6 |
|  | ABLUP-A |  | FAMK |  |  |  | MET | -6773.5 | 13761.0 | 13797.49 | 7 |
|  | **ABLUP-A** |  | **CS+DIAG** |  |  |  | **MET** | **-6873.47** | **13760.95** | **13797.49** | **7** |
|  | ABLUP-AD |  | CS+DIAG | IDEN |  |  | MET | -6872.07 | 13760.14 | 13801.90 | 8 |
|  | **ABLUP-AD** |  | **CS+DIAG** | **DIAG** |  |  | **MET** | **-6868.92** | **13755.85** | **13802.83** | **9** |
|  | ABLUP-AD |  | CS+DIAG | CS |  |  | MET | -6869.94 | 13757.88 | 13804.86 | 9 |
|  | GBLUP-A |  | IDEN |  |  |  | ST | -6879.78 | 13769.57 | 13795.67 | 5 |
|  | GBLUP-A |  | DIAG |  |  |  | ST | -6875.11 | 13762.21 | 13793.53 | 6 |
|  | GBLUP-A |  | CS |  |  |  | MET | -6875.42 | 13762.84 | 13794.16 | 6 |
|  | GBLUP-A |  | FAMK |  |  |  | MET | -6874.05 | 13762.10 | 13798.64 | 7 |
|  | **GBLUP-A** |  | **CS+DIAG** |  |  |  | **MET** | **-6874.05** | **13762.10** | **13798.64** | **7** |
|  | **GBLUP-AD** |  | **CS+DIAG** | **IDEN** |  |  | **MET** | **-6870.21** | **13756.42** | **13798.18** | **8** |
|  | GBLUP-AD |  | CS+DIAG | DIAG |  |  | MET | -6870.66 | 13759.32 | 13806.30 | 9 |
|  | GBLUP-AD |  | CS+DIAG | CS |  |  | MET | -6870.08 | 13758.15 | 13805.14 | 9 |
|  | GBLUP-ADE |  | CS+DIAG | IDEN | IDEN-$G_{aa}$ |  | MET | -6870.21 | 13758.42 | 13805.41 | 9 |
|  | GBLUP-ADE |  | CS+DIAG | IDEN | IDEN-$G_{ad}$ |  | MET | -6870.21 | 13758.42 | 13805.41 | 9 |
|  | GBLUP-ADE |  | CS+DIAG | IDEN | IDEN-$G_{dd}$ |  | MET | -6870.21 | 13758.42 | 13805.41 | 9 |
|  | GBLUP-ADE |  | CS+DIAG | IDEN | IDEN-G3* |  | MET | -6870.21 | 13762.42 | 13819.85 | 11 |
| Pilodyn | ABLUP-A |  | IDEN |  |  |  | ST | -1729.70 | 3469.40 | 3495.50 | 5 |
|  | ABLUP-A |  | DIAG |  |  |  | ST | -1739.93 | 3491.87 | 3523.19 | 6 |
|  | **ABLUP-A** |  | **CS** |  |  |  | **MET** | **-1727.77** | **3467.55** | **3498.87** | **6** |
|  | ABLUP-A |  | FAMK |  |  |  | MET | -1727.77 | 3469.55 | 3506.08 | 7 |
|  | ABLUP-A |  | CS+DIAG |  |  |  | MET | -1727.77 | 3469.55 | 3506.08 | 7 |
|  | **ABLUP-AD** |  | **CS** | **IDEN** |  |  | **MET** | **-1727.77** | **3469.55** | **3506.09** | **7** |
|  | ABLUP-AD |  | CS | DIAG |  |  | MET | -1727.63 | 3471.26 | 3513.02 | 8 |
|  | ABLUP-AD |  | CS | CS |  |  | MET | -1727.68 | 3471.36 | 3513.11 | 8 |
|  | **GBLUP-A** |  | **IDEN** |  |  |  | **ST** | **-1737.44** | **3484.88** | **3510.97** | **5** |
|  | GBLUP-A |  | DIAG |  |  |  | ST | -1748.95 | 3509.89 | 3541.21 | 6 |
|  | GBLUP-A |  | CS |  |  |  | MET | -1736.77 | 3485.54 | 3516.86 | 6 |
|  | GBLUP-A |  | FAMK |  |  |  | MET | -1736.71 | 3487.43 | 3523.96 | 7 |
|  | GBLUP-A |  | CS+DIAG |  |  |  | MET | -1736.71 | 3487.43 | 3523.96 | 7 |
|  | GBLUP-AD |  | IDEN | IDEN |  |  | MET | -1737.44 | 3486.88 | 3518.19 | 6 |
|  | **GBLUP-AD** |  | **IDEN** | **DIAG** |  |  | **MET** | **-1735.87** | **3485.74** | **3522.27** | **7** |
|  | GBLUP-AD |  | IDEN | CS |  |  | MET | -1737.22 | 3488.44 | 3524.98 | 7 |
|  | GBLUP-ADE |  | IDEN | IDEN | IDEN-$G_{aa}$ |  | MET | -1737.44 | 3488.88 | 3525.41 | 7 |
|  | GBLUP-ADE |  | IDEN | IDEN | IDEN-$G_{ad}$ |  | MET | -1737.44 | 3488.88 | 3525.41 | 7 |
|  | GBLUP-ADE |  | IDEN | IDEN | IDEN-$G_{dd}$ |  | MET | -1737.44 | 3488.88 | 3525.41 | 7 |
|  | GBLUP-ADE |  | IDEN | IDEN | IDEN-*G*3 |  | MET | -1736.77 | 3493.54 | 3545.74 | 10 |
| Velocity | ABLUP-A |  | IDEN |  |  |  | ST | 1188.92 | -2367.84 | -2341.79 | 5 |
|  | ABLUP-A |  | DIAG |  |  |  | ST | 1178.02 | -2344.05 | -2312.79 | 6 |
|  | **ABLUP-A** |  | **CS** |  |  |  | **MET** | **1192.66** | **-2373.33** | **-2342.07** | **6** |
|  | ABLUP-A |  | FAMK |  |  |  | MET | 1192.79 | -2371.59 | -2335.12 | 7 |
|  | ABLUP-A |  | DIAG |  |  |  | MET | 1192.79 | -2371.59 | -2335.12 | 7 |
|  | **ABLUP-AD** |  | **CS** | **IDEN** |  |  | **MET** | **1194.59** | **-2375.19** | **-2338.72** | **7** |
|  | ABLUP-AD |  | CS | DIAG |  |  | MET | 1193.04 | -2370.08 | -2328.40 | 8 |
|  | ABLUP-AD |  | CS | CS |  |  | MET | 1194.59 | -2373.19 | -2331.51 | 9 |
|  | GBLUP-A |  | IDEN |  |  |  | ST | 1178.80 | -2347.59 | -2321.54 | 5 |
|  | GBLUP-A |  | DIAG |  |  |  | ST | 1172.79 | -2333.59 | -2302.32 | 6 |
|  | **GBLUP-A** |  | **CS** |  |  |  | **MET** | **1183.37** | **-2354.73** | **-2323.47** | **6** |
|  | GBLUP-A |  | FAMK |  |  |  | MET | 1184.03 | -2354.06 | -2317.59 | 7 |
|  | GBLUP-A |  | CS+DIAG |  |  |  | MET | 1184.03 | -2354.06 | -2317.59 | 7 |
|  | **GBLUP-AD** |  | **CS** | **IDEN** |  |  | **MET** | **1184.63** | **-2355.26** | **-2318.79** | **7** |
|  | GBLUP-AD |  | CS | DIAG |  |  | MET | 1184.39 | -2352.77 | -2311.09 | 8 |
|  | GBLUP-AD |  | CS | CS |  |  | MET | 1184.63 | -2353.26 | -2311.58 | 8 |
|  | GBLUP-ADE |  | CS | IDEN | IDEN-$G_{aa}$ |  | MET | 1184.66 | -2353.32 | -2311.64 | 8 |
|  | GBLUP-ADE |  | CS | IDEN | IDEN-$G_{ad}$ |  | MET | 1184.63 | -2353.26 | -2311.58 | 8 |
|  | GBLUP-ADE |  | CS | IDEN | IDEN-$G_{dd}$ |  | MET | 1184.64 | -2353.29 | -2311.60 | 8 |
|  | GBLUP-ADE |  | CS | IDEN | IDEN-G3 |  | MET | 1184.66 | -2349.32 | -2297.22 | 10 |
| MOE | ABLUP-A |  | IDEN |  |  |  | ST | -2350.00 | 4709.99 | 4736.04 | 5 |
|  | ABLUP-A |  | DIAG |  |  |  | ST | -2360.56 | 4733.12 | 4764.37 | 6 |
|  | **ABLUP-A** |  | **CS** |  |  |  | **MET** | **-2347.46** | **4706.92** | **4738.18** | **6** |
|  | ABLUP-A |  | FAMK |  |  |  | MET | -2346.96 | 4707.91 | 4744.38 | 7 |
|  | ABLUP-A |  | CS+DIAG |  |  |  | MET | -2346.98 | 4707.96 | 4744.42 | 7 |
|  | **ABLUP-AD** |  | **CS** | **IDEN** |  |  | **MET** | **-2347.46** | **4708.92** | **4745.39** | **7** |
|  | ABLUP-AD |  | CS | DIAG |  |  | MET | -2346.89 | 4709.78 | 4751.45 | 8 |
|  | ABLUP-AD |  | CS | CS |  |  | MET | -2346.89 | 4711.78 | 4758.66 | 9 |
|  | GBLUP-A |  | IDEN |  |  |  | ST | -2359.51 | 4729.01 | 4755.06 | 5 |
|  | GBLUP-A |  | DIAG |  |  |  | ST | -2370.45 | 4752.90 | 4784.16 | 6 |
|  | **GBLUP-A** |  | **CS** |  |  |  | **MET** | **-2357.84** | **4727.67** | **4758.93** | **6** |
|  | GBLUP-A |  | FAMK |  |  |  | MET | -2357.41 | 4728.82 | 4765.29 | 7 |
|  | GBLUP-A |  | CS+DIAG |  |  |  | MET | -2357.41 | 4728.82 | 4765.29 | 7 |
|  | **GBLUP-AD** |  | **CS** | **IDEN** |  |  | **MET** | **-2357.84** | **4729.67** | **4766.14** | **7** |
|  | GBLUP-AD |  | CS | DIAG |  |  | MET | -2357.19 | 4730.38 | 4772.06 | 8 |
|  | GBLUP-AD |  | CS | CS |  |  | MET | -2357.84 | 4731.67 | 4773.35 | 8 |
|  | GBLUP-ADE |  | CS | IDEN | IDEN-$G_{aa}$ |  | MET | -2357.84 | 4731.67 | 4773.35 | 8 |
|  | GBLUP-ADE |  | CS | IDEN | IDEN-$G_{ad}$ |  | MET | -2357.84 | 4731.67 | 4773.35 | 8 |
|  | GBLUP-ADE |  | CS | IDEN | IDEN-$G_{dd}$ |  | MET | -2357.84 | 4731.67 | 4773.35 | 8 |
|  | GBLUP-ADE |  | CS | IDEN | IDEN_G3 |  | MET | -2357.84 | 4735.67 | 4787.77 | 10 |

Variance and covariance structures: IDEN, identity; DIAG, Diagonal; CS, compound symmetry; FAMK, a factor analytic with the main marker/genetic term and k factors;.* G3 represents GBLUP-ADE model including three first-order epistatic effects (i.e. the random additive by additive epistatic effects, additive by dominance epistatic effects, and dominance by dominance epistatic effects). No. is the number of variance parameters. Bold means the best model in ABLUP-A, ABLUP-AD, GBLUP-A, and GBLUP-AD.

**Table S2**. Estimates of variance components (VC), their standard errors (SE) and the variance proportion at each site for Pilodyn and MOE from the five genetic models fitted (ABLUP-A, ABLUP-AD, GBLUP-A, GBLUP-AD, and GBLUP-ADE)

| Trait | VC | ABLUP-A | |  | ABLUP-AD | | | |  | | GBLUP-A | | | |  | | GBLUP-AD | | | |  | | GBLUP-ADE | | |
| --- | --- | --- | --- | --- | --- | --- | --- | --- | --- | --- | --- | --- | --- | --- | --- | --- | --- | --- | --- | --- | --- | --- | --- | --- | --- |
|  |  | Value (SE) | % |  | Value (SE) | | % | |  | | Value (SE) | | % | |  | | Value (SE) | | % | |  | | Value (SE) | | % |
| Pilodyn | $\sigma_{b_{1}}^{2}$ | 0.24 (0.14) | 4.2 |  | 0.24 (0.14) | | 4.2 | |  | | 0.23 (0.13) | | 4.2 | |  | | 0.23 (0.13) | | 4.2 | |  | | 0.23 (0.13) | | 4.2 |
|  | $\sigma_{b_{2}}^{2}$ | 0.63 (0.24) | 11.1 |  | 0.63 (0.24) | | 11.1 | |  | | 0.71 (0.26) | | 13 | |  | | 0.71 (0.26) | | 13 | |  | | 0.71 (0.26) | | 13.0 |
|  | $\sigma_{a_{1}}^{2}$ | 2.30 (0.57) | 40.1 |  | 2.30 (0.57) | | 40.1 | |  | | 1.79 (0.34) | | 32 | |  | | 1.79 (0.34) | | 32 | |  | | 1.79 (0.34) | | 32.0 |
|  | $\sigma_{a_{12}}^{2}$ | 2.03 (0.19) |  |  | 2.03 (0.19) | |  | |  | | 1.61 (0.35) | |  | |  | | 1.61 (0.35) | |  | |  | | 1.61 (0.35) | |  |
|  | $\sigma_{a_{2}}^{2}$ | 2.30 (0.57) | 40.7 |  | 2.30 (0.57) | | 40.7 | |  | | 1.79 (0.34) | | 32.8 | |  | | 1.79 (0.34) | | 32.8 | |  | | 1.79 (0.34) | | 32.8 |
|  | $\sigma_{d_{1}}^{2}$ |  |  |  | 0 (0) | | 0 | |  | |  | |  | |  | | 0 (0) | | 0 | |  | | 0 (0) | | 0 |
|  | $\sigma_{d_{2}}^{2}$ |  |  |  | 0 (0) | | 0 | |  | |  | |  | |  | | 0 (0) | | 0 | |  | | 0 (0) | | 0 |
|  | $\sigma_{aa}^{2}$ |  |  |  |  | |  | |  | |  | |  | |  | |  | |  | |  | | 0 (0) | | 0 |
|  | $\sigma_{ad}^{2}$ |  |  |  |  | |  | |  | |  | |  | |  | |  | |  | |  | | 0 (0) | | 0 |
|  | $\sigma_{dd}^{2}$ |  |  |  |  | |  | |  | |  | |  | |  | |  | |  | |  | | 0 (0) | | 0 |
|  | $\sigma_{e_{1}}^{2}$ | 3.20 (0.41) | 55.7 |  | 3.20 (0.41) | | 55.7 | |  | | 0.32 (3.57) | | 63.9 | |  | | 3.57 (0.32) | | 63.9 | |  | | 3.57 (0.32) | | 63.9 |
|  | $\sigma_{e_{2}}^{2}$ | 2.72 (0.36) | 48.2 |  | 2.72 (0.36) | | 48.2 | |  | | 0.27 (2.96) | | 54.2 | |  | | 2.96 (0.27) | | 54.2 | |  | | 2.96 (0.27) | | 54.2 |
|  | $h_{1}^{2}$ | 0.37 (0.09) |  |  | 0.37 (0.09) | |  | |  | | 0.06 (0.30) | |  | |  | | 0.30 (0.06) | |  | |  | | 0.30 (0.06) | |  |
|  | $h_{2}^{2}$ | 0.40 (0.09) |  |  | 0.40 (0.09) | |  | |  | | 0.06 (0.34) | |  | |  | | 0.34 (0.06) | |  | |  | | 0.34 (0.06) | |  |
|  | $H_{1}^{2}$ |  |  |  | 0.37 (0.09) | |  | |  | |  | |  | |  | | 0.30 (0.06) | |  | |  | | 0.30 (0.06) | |  |
|  | $H_{2}^{2}$ |  |  |  | 0.40 (0.09) | |  | |  | |  | |  | |  | | 0.34 (0.06) | |  | |  | | 0.34 (0.06) | |  |
|  |  |  |  |  |  | |  | |  | |  | |  | |  | |  | |  | |  | |  | |  |
| MOE | $\sigma_{b_{1}}^{2}$ | 0.49 (0.31) | 3.0 |  | 0.49 (0.31) | 3.0 | |  | | 0.45 (0.30) | | 2.8 | |  | | 0.45 (0.30) | | 2.8 | |  | | 0.45 (0.30) | | 2.8 | |
|  | $\sigma_{b_{2}}^{2}$ | 0.68 (0.31) | 5.1 |  | 0.68 (0.31) | 5.1 | |  | | 0.87 (0.37) | | 6.8 | |  | | 0.87 (0.37) | | 6.8 | |  | | 0.87 (0.37) | | 6.8 | |
|  | $\sigma_{a_{1}}^{2}$ | 6.63 (1.61) | 40.4 |  | 6.63 (1.61) | 40.4 | |  | | 5.23 (0.94) | | 32.9 | |  | | 5.23 (0.94) | | 32.9 | |  | | 5.23 (0.94) | | 32.9 | |
|  | $\sigma_{a_{12}}^{2}$ | 5.82 (1.60) |  |  | 5.82 (1.60) |  | |  | | 4.54 (0.95) | |  | |  | | 4.54 (0.95) | |  | |  | | 4.54 (0.95) | |  | |
|  | $\sigma_{a_{2}}^{2}$ | 6.63 (1.61) | 50 |  | 6.63 (1.61) | 50 | |  | | 5.23 (0.94) | | 41.1 | |  | | 5.23 (0.94) | | 41.1 | |  | | 5.23 (0.94) | | 41.1 | |
|  | $\sigma_{d_{1}}^{2}$ |  |  |  | 0 (0) | 0 | |  | |  | |  | |  | | 0 (0) | | 0 | |  | | 0 (0) | | 0 | |
|  | $\sigma_{d_{2}}^{2}$ |  |  |  | 0 (0) | 0 | |  | |  | |  | |  | | 0 (0) | | 0 | |  | | 0 (0) | | 0 | |
|  | $\sigma_{aa}^{2}$ |  |  |  |  |  | |  | |  | |  | |  | |  | |  | |  | | 0 (0) | | 0 | |
|  | $\sigma_{ad}^{2}$ |  |  |  |  |  | |  | |  | |  | |  | |  | |  | |  | | 0 (0) | | 0 | |
|  | $\sigma_{dd}^{2}$ |  |  |  |  |  | |  | |  | |  | |  | |  | |  | |  | | 0 (0) | | 0 | |
|  | $\sigma_{e_{1}}^{2}$ | 9.29 (0.41) | 56.6 |  | 9.29 (1.17) | 56.6 | |  | | 10.24 (0.92) | | 64.3 | |  | | 10.24 (0.92) | | 64.3 | |  | | 10.24 (0.92) | | 64.3 | |
|  | $\sigma_{e_{2}}^{2}$ | 5.96 (0.36) | 44.9 |  | 5.96 (0.95) | 44.9 | |  | | 6.61 (0.68) | | 52.0 | |  | | 6.61 (0.68) | | 52.0 | |  | | 6.61 (0.68) | | 52.0 | |
|  | $h_{1}^{2}$ | 0.37 (0.09) |  |  | 0.37 (0.09) |  | |  | | 0.29 (0.05) | |  | |  | | 0.29 (0.05) | |  | |  | | 0.29 (0.05) | |  | |
|  | $h_{2}^{2}$ | 0.46 (0.10) |  |  | 0.46 (0.10) |  | |  | | 0.38 (0.07) | |  | |  | | 0.38 (0.07) | |  | |  | | 0.38 (0.07) | |  | |
|  | $H_{1}^{2}$ |  |  |  | 0.37 (0.09) |  | |  | |  | |  | |  | | 0.29 (0.05) | |  | |  | | 0.29 (0.05) | |  | |
|  | $H_{2}^{2}$ |  |  |  | 0.46 (0.10) |  | |  | |  | |  | |  | | 0.38 (0.07) | |  | |  | | 0.38 (0.07) | |  | |

Notes:$\sigma_{b_{1}}^{2}$and $\sigma_{b_{2}}^{2}$ are the block variance for site 1 and site 2. $\sigma_{a1}^{2}$ $\sigma_{a2}^{2}$, and $\sigma_{a12}$ are the additive variances for site 1, site 2 and additive covariance between site 1 and site 2, respectively. $\sigma_{d1}^{2}$ $\sigma_{d2}^{2}$, and $\sigma_{d12}$ are the dominance variances for site 1, site 2 and dominance covariance between site 1 and site 2.$\sigma_{aa}^{2}$, $\sigma_{ad}^{2}$, and $\sigma_{dd}^{2}$ are the additive × additive epistatic variance, additive × dominance epistatic variance, and dominance × dominance epistatic variances, respectively. $\sigma_{e1}^{2}$ and $\sigma_{e2}^{2}$ are the residual variances for site 1 and site 2, respectively. $h_{1}^{2}$ and $h_{2}^{2}$ are the narrow-sense heritability for site 1 and site 2, respectively. $H_{1}^{2}$ and $H_{2}^{2}$ are the broad-sense heritability for site 1 and site 2, respectively.

**Table S3.** Response to genomic selection (RGS), including three different selection scenarios, which were based on 1) only main marker effects (M), 2) M + marker-by-environment interaction effects (A), and 3) A + dominance effects (AD) from GBLUP-AD and ABLUP-AD models for tree height, Pilodyn, velocity, and MOE expressed as a percentage gain of the average population mean per year, compared with response to conventional phenotypic selection (RPS) per year also including dominance effect (ABLUP-AD) calculated for the top 50 individuals selected by three different GS scenarios.

| **Trait** | **Site** | **Model** | **Effects** | **Gain %/year** | **Increase (%)** |
| --- | --- | --- | --- | --- | --- |
| Tree height | Site 1 | ABLUP-AD | AD | 0.26 |  |
|  | Site 1 | GBLUP-AD | AD | 0.43 | 64.92 |
|  | Site 1 | GBLUP-AD | A | 0.41 | 56.30 |
|  | Site 1 | GBLUP-AD | M | 0.41 | 58.42 |
|  | **Site 2** | ABLUP-AD | **AD** | **0.32** |  |
|  | **Site 2** | GBLUP-AD | **AD** | **0.54** | **68.87** |
|  | **Site 2** | GBLUP-AD | **A** | **0.45** | **41.24** |
|  | **Site 2** | GBLUP-AD | **M** | **0.46** | **43.68** |
| Pilodyn | Site 1 | ABLUP-AD | AD | 0.45 |  |
|  | Site 1 | GBLUP-AD | AD | 0.83 | 84.18 |
|  | Site 1 | GBLUP-AD | A | 0.83 | 84.18 |
|  | Site 1 | GBLUP-AD | M | 0.82 | 83.31 |
|  | **Site 2** | **ABLUP-AD** | **AD** | **0.44** |  |
|  | **Site 2** | **GBLUP-AD** | **AD** | **0.80** | **81.33** |
|  | **Site 2** | **GBLUP-AD** | **A** | **0.80** | **81.33** |
|  | **Site 2** | **GBLUP-AD** | **M** | **0.80** | **81.46** |
| Velocity | Site 1 | ABLUP-AD | AD | 0.22 |  |
|  | Site 1 | GBLUP-AD | AD | 0.42 | 91.29 |
|  | Site 1 | GBLUP-AD | A | 0.41 | 86.92 |
|  | Site 1 | GBLUP-AD | M | 0.39 | 78.92 |
|  | **Site 2** | **ABLUP-AD** | **AD** | **0.22** |  |
|  | **Site 2** | **GBLUP-AD** | **AD** | **0.41** | **88.17** |
|  | **Site 2** | **GBLUP-AD** | **A** | **0.40** | **82.89** |
|  | **Site 2** | **GBLUP-AD** | **M** | **0.40** | **80.75** |
| MOE | Site 1 | ABLUP-AD | AD | 0.50 |  |
|  | Site 1 | GBLUP-AD | AD | 0.96 | 92.58 |
|  | Site 1 | GBLUP-AD | A | 0.96 | 92.58 |
|  | Site 1 | GBLUP-AD | M | 0.95 | 90.33 |
|  | **Site 2** | **ABLUP-AD** | **AD** | **0.58** |  |
|  | **Site 2** | **GBLUP-AD** | **AD** | **1.10** | **89.90** |
|  | **Site 2** | **GBLUP-AD** | **A** | **1.10** | **89.90** |
|  | **Site 2** | **GBLUP-AD** | **M** | **1.08** | **86.66** |


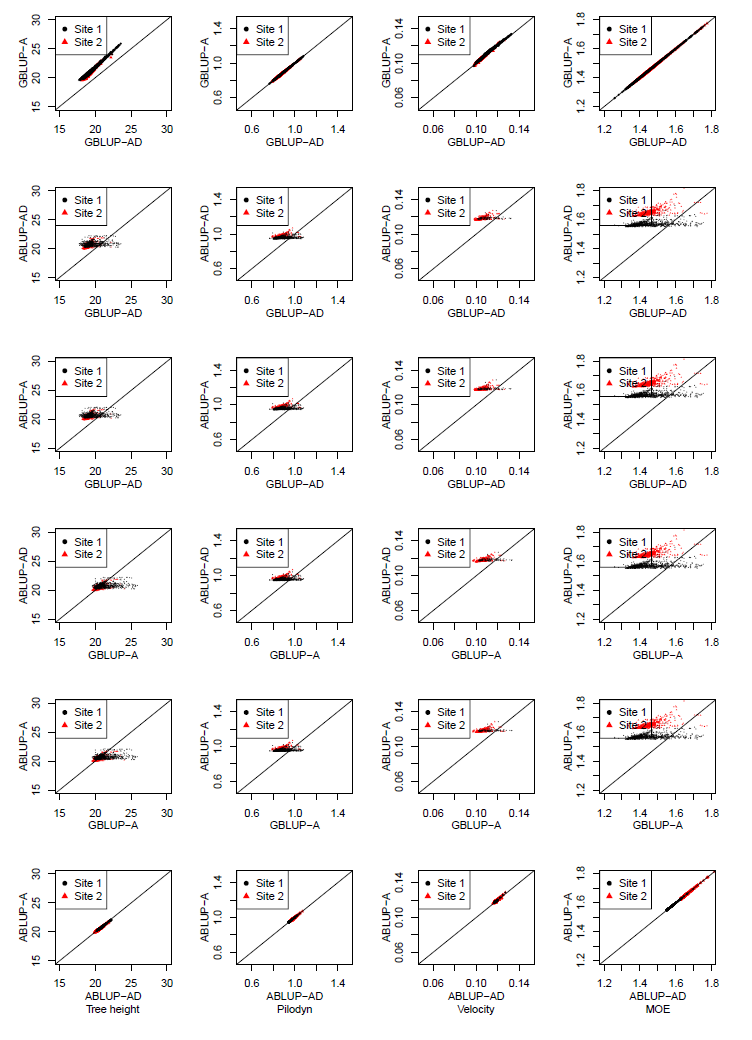


**Figure S1.** Standard errors for the predictions (SEP) for breeding values comparisons between GBLUP-AD and GBLUP-A, between GBLUP-AD and ABLUP-AD, between GBLUP-AD and ABLUP-A, between GBLUP-A and ABLUP-AD, between GBLUP-A and ABLUP-A, and between ABLUP-AD and ABLUP-A for tree height, Pilodyn, velocity and MOE.


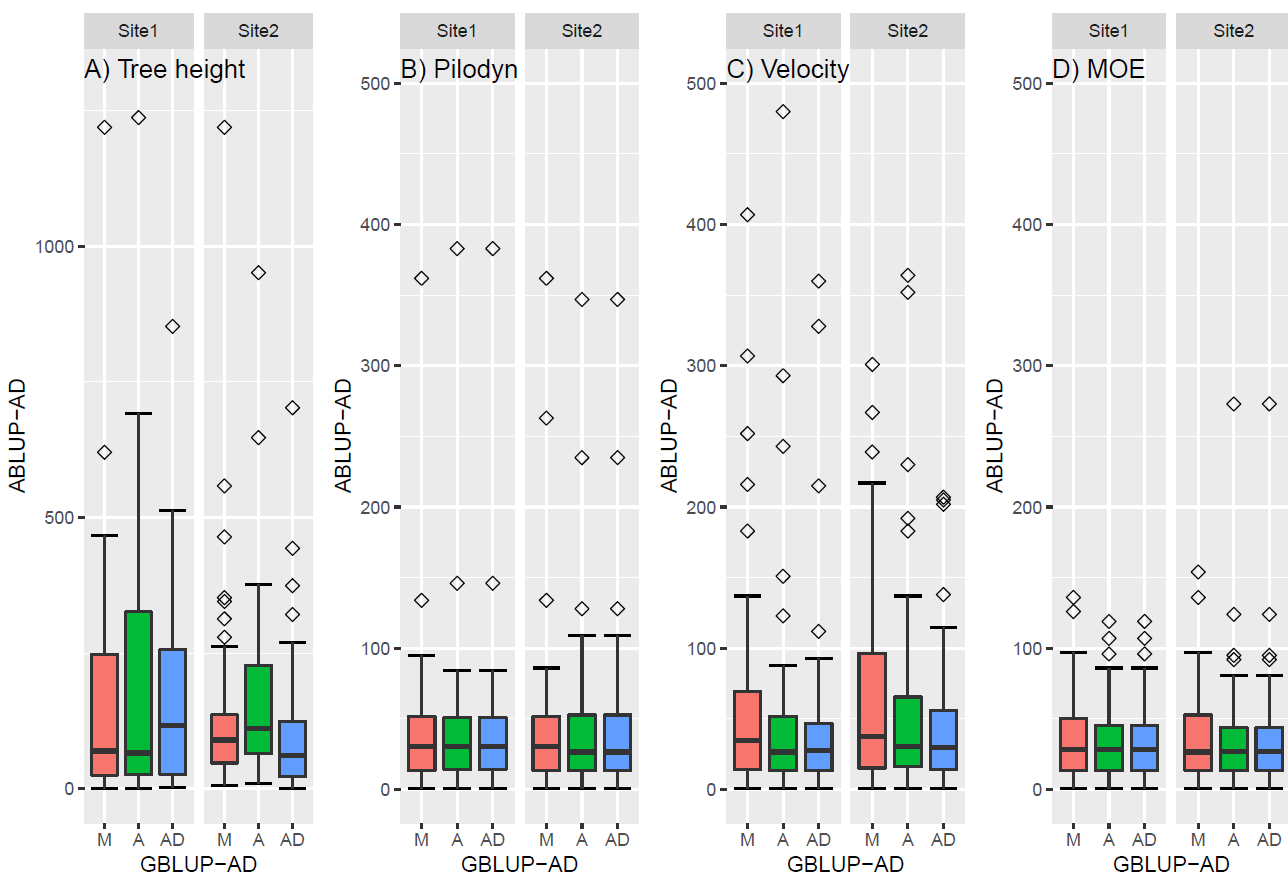


**Figure S2.** Boxplots of the genomic-based expected genetic values (GEGV) of the top 50 selected individuals, scaled to the total expected genetic value (EGV) ranking of all individuals in site 1 and site 2. Three different selection scenarios for the selection of the top 50 individuals including selections based on 1) only main marker effects (M), 2) main marker effects plus genotype-by-environment interaction effects (A), and 3) A plus dominance (AD) from GBLUP-AD model for A) tree height, B) Pilodyn, C) velocity, and D) MOE.
